# Anthropometric and metabolic differences and distribution of *ABCG2 rs2231142* variant between lowland and highland Papuans in West Papua, Indonesia

**DOI:** 10.1186/s40101-025-00394-7

**Published:** 2025-05-20

**Authors:** Ferry Fredy Karwur, Monica Hermina Sharon Otline Yocku, Debby Agustin Enoch, Rambu Lawu Nedi Kristanti Retno Triandhini, Venti Agustina, Meyga Feybbi Lakukua, Ferdy Semuel Rondonuwu, Jerry Ferry Langkun

**Affiliations:** 1 Faculty of Health Sciences, Satya Wacana Christian University, Salatiga, Central Java 50711 Indonesia; 2 Molecular Biology Laboratory-BSL3, Satya Wacana Christian University, Salatiga, Central Java 50714 Indonesia; 3 Faculty of Science and Mathematics, Satya Wacana Christian University, Salatiga, Central Java 50711 Indonesia

**Keywords:** West Papua, Metabolic adaptation, Asian phenotype, *ABCG2*

## Abstract

**Background:**

Papuan people inhabiting the island of New Guinea are the most ancient population living outside Africa, having resided in the region for at least 50,000 years. The arrival of Austronesian speakers and other group from mainland Asia around 3000 years or so created a peculiar genetic mixture, particularly in lowland/coastal areas. We investigated the anthropometric and blood chemical differences alongside the population structure of the *ABCG2 rs2231142* genetic variant of West Papuans from lowland/coastal and highland areas to understand metabolic risk differences between these two populations.

**Results:**

We studied West Papuan students from lowland/coastal areas (*n* = 78, 45 males, 33 females) and from highland areas (*n* = 65, 40 males, 25 females). We found the following:

1. The lowland/coastal Papuans were taller, with lower BMI, central obesity, and triceps. Contrarily, highland Papuans have a more gynoid body shape, with higher WC, HC, WHR, and WHtR. The skinfolds were significantly thicker in women from the highlands.
2. There was actually a negative correlation between BMI and central adiposity with UA and FBG to those from the highlands. The lowland/coastal Papuans indicated an Asian-type metabolic traits, with higher fasting glucose levels at lower BMI and lower central adiposity.
3. UA concentration and DBP were strongly correlated with obesity of the Papuans from lowlands/coasts and not in the Papuans from highlands.
4. There was a striking difference in the *ABCG2 rs2231142* > T allele frequency in those from the lowlands/coasts (22%) compared to those from the highlands of West Papua (7%). The T variant in the latter is all heterozygous.

**Conclusions:**

The higher adiposity and thicker skinfolds observed in highland Papuans are thought to be adaptive responses to the high-altitude environment, enabling greater adipose tissue expandability and energy storage capacity while maintaining metabolic homeostasis. In contrast, the lowland/coastal Papuans exhibit an Asian metabolic phenotype, which is more prone to metabolic derangements at lower adiposity. Our findings on the population distribution of the *ABCG2 rs2231142* > *T* variant support the idea that its presence in the Papuan highlands is through demic diffusion of the variant from ISEA, indicating that the two populations are separate entities displaying differences in metabolic risks.

**Supplementary Information:**

The online version contains supplementary material available at 10.1186/s40101-025-00394-7.

## Background

The world is currently facing a pandemic of noncommunicable diseases, especially metabolic disorders, including obesity, type 2 diabetes mellitus (T2DM), [1]. The spread of these diseases at a global scale occurs through the interaction of factors that operate globally (modernization, global trade) with demographic factors and local drivers [1–3] affecting diet and lifestyle. Austronesian-speaking peoples, spread from Formosa Island on the east coast of China to New Zealand and Madagascar, have a number of cardio-metabolic disorders with extreme degrees. Aboriginal Taiwanese, Filipinos, Minahasans, New Zealand’s indigenous Maori, and Pacific Islanders, all of whom speak Austronesian languages, have been reported to have the highest prevalence of hyperuricemia and gout in the world [4]. Likewise, Oceanian islanders, especially Micronesians and Polynesians, have the highest prevalence rates of obesity and T2DM in the world [5]. High levels of endogenous hyperuricemia are thought to be a predisposing factor for the development of obesity and T2DM in the context of a Western lifestyle and diet [6]. A complex history of migration has led to genetic/epigenetic factors, local environment, and modern lifestyle influencing the incidence and distribution of these diseases across this vast region.

Papua is a region that geographically and demographically intersects and interacts with Micronesians and Polynesians and belongs to what is so-called Melanesia, “the island of black people” [7]. The Papuan consists of two races whose existence was formed by a unique history of settlement and migration in the region. The Papuan highlanders are the oldest population in the world outside Africa and have inhabited the region for around 50,000 to 65,000 years [8]. They lived in small, interconnected populations, as illustrated in their languages with their common roots, agricultural practices, and Pleistocene lithic culture [9]. They have adapted and been selected to this specific niche, including pathogens and natural resources [10]. On the other hand, the Papuans living in lowland/coastal areas have changed radically with the arrival of Austronesian speakers and other groups from mainland Asia through Taiwan and Eastern Indonesia for at least 3000 years or so [11, 12]. The newcomers brought a new culture, the Neolithic culture, including languages, technologies, material culture, and agriculture [13]. Genetic introgression occurs mainly in the region and creates a genetic hybrid zone [8, 14]. Furthermore, the Neandertals and Denisovan genetic divergence and adaptation within the Papuan population added to the complexity [15].

The existence of the Papuan population, with its prehistoric background and unique physical and social diversification, provides an opportunity to study the fundamental factors that reveal the workings of global influences and local exceptionality in causing metabolic diseases. Our long-term goal is to understand the genetic construction that plays a significant role in the emergence of metabolic diseases, in the highly diverse racial and ethnic backgrounds of Indonesia, including West Papua. The aim of the study we report here was to compare the anthropometric profiles and clinical chemistry relevant to the emergence of obesity-related metabolic diseases, and the population structure of the *ABCG2 rs2231142* variant — one of the prominent genetic variants for hyperuricemia (HUA) in an East Asian population [16] and tophaceous gout of Austronesian ancestry [17]. The *ABCG2 rs2231142* variant could be a genetic marker of East Asian genetic penetrance to the Papuan population and might delineate the West Papua lowland/coastal population from the West Papua highland population. We proposed that the West Papua highlanders and West Papua lowland/coastal peoples are unique genetic entities suitable for uncovering genetic variants relevant to the study of the interaction between genes and the environment involving in obesity-related metabolic diseases and metabolic dysfunctions. To test this idea, we compared Satya Wacana Christian University students from the West Papua highlands to those from West Papua lowlands/coasts.

## Methods

### Study design, time, and place

This is a cross-sectional observational study of anthropometric, clinical, blood chemistry, and genetic profiles of the *ABCG2 rs2231142* variant of lowland and highland Papuan students studying at Satya Wacana Christian University. Data collection was conducted from March 2018 to January 2019 in Salatiga. Laboratory analysis was carried out in the Faculty of Medicine and Health Sciences, Molecular Biology Laboratory-BSL3 of Satya Wacana, and PT Enigma Saintia Solusindo, Tangerang.

### Research participants

In 2018, there were 770 students from various regions of West Papua studying at Satya Wacana. Based on their family names, places of origin, and languages, we identified 377 students as ethnically Papuan, grouping them into highland Papuans and lowland Papuans. The research participants are those able to communicate fluently in Indonesian and have lived in Salatiga for at least 6 months. Participants from the lowland/coastal areas who agreed to participate in this study came from 12 different regions and ethnicities and spoke 15 different languages. Highland participants, whose parents came from the same tribal background, represented 11 tribes. This group also included four students from inter-tribal marriages within the Papuan highlands, while those born to parents from both the Papuan highlands and lowlands were excluded. We conducted door-to-door visits to their residences, meeting participants either in groups or individually to explain the purpose of the study and the nature of their involvement in the research. We also verified the tribal affiliations of participants’ parents to confirm their ethnic identities. A total of 65 Papuan highlander students (40 men and 25 women) and 78 Papuan lowlander/coastal students (45 men and 33 women) agreed to participate and provided informed consent.

### Data collections

We collected data through questionnaires and interviews, gathering information that includes name, sex, age, ethnic origin, personal and family history of joint pain, and length of residence in Salatiga. We measured anthropometric information [body height (BH), body weight (BW), waist circumference (WC), hip circumference (HC) [18], and skinfold (by measuring of biceps, triceps, subscapular, and suprailiac)] and clinical chemistry data [diastolic blood pressure (DBP) and systolic blood pressure (SBP), uric acid (UA) concentration, random blood glucose (RBG), fasting blood glucose (FBG), and total cholesterol (TC)].

#### Body height

BH was measured using a microtoise with a 200-cm scale and an accuracy of 0.1 cm. During the measurement, participants stood upright against a wall, and the microtoise headpiece was lowered to touch the top of the head; the value displayed on the scale was recorded as the participant’s height.

#### Body weight

BW was measured using a *OneMed Elegance* scale with a capacity of 120 kg and an accuracy level of 0.1 kg. The scale was calibrated to zero before each measurement, after which the participant stepped onto the scale, and the displayed weight was recorded.

#### Waist circumference

The WC was measured to the nearest 0.1 cm using a flexible nonelastic measuring tape, which is done by measuring the circumference of the abdomen in between the middle of the crista iliac with the bottom rib, horizontally.

#### Hip circumference

The HC was measured to the nearest 0.1 cm using a flexible nonelastic measuring tape, which is done by measuring the largest circumference around the buttocks.

#### Skinfold

Skinfolds were obtained from fat measurements in the biceps, triceps, subscapular, and suprailiac areas, using a skinfold caliper in millimeters (mm).

### Calculations

BMI was counted by dividing weight (kg) by height (m^2^). Waist-to-hip ratio (WHR) was estimated by dividing waist circumference by hip circumference. Waist-to-height ratio (WHtR) was calculated by dividing the size of the waist circumference with the circumference of the abdomen. The WHtR was calculated by dividing WC by height. Fat percentage was calculated using the Durnin-Womerslay (1974) formula and differentiated according to sex.

### Clinical and blood chemistry

Clinical measurements consisting of diastolic and systolic blood pressure, and blood chemistry, including UA, FBG, RBG, and TC, were performed using a rapid peripheral blood test. Blood pressure was monitored using an *OMRON Automatic Blood Pressure Monitor*. In addition, peripheral blood of UA, FBG, and TC was measured using the *Nesco MultiCcheck 3in1 Multifunction Monitoring System* obtained by fingertip puncture. FBG tests were examined in the morning after an overnight fast for at least 8 h.

### Blood collection and DNA isolation

Blood sampling was carried out by visiting respondent’s door to door, collected by a certified nurse using a 5-mL syringe through a vein with a volume of 3 mL. The blood was immediately transferred to EDTA K2 tubes, placed in a cooler filled with ice, transported to the Molecular Biology Laboratory-BSL3, and stored in a freezer at − 20 °C. Deoxyribonucleic acid (DNA) isolation of all blood samples was carried out using the Genomic DNA Mini Kit: Blood/Cultured Cell (Geneaid, no. cat: GB100/300) according to the manufacturer’s protocol. The DNA results were visualized using agarose gel electrophoresis and a UV Transilluminator. The DNA concentration was quantified using UV–VIS at a wavelength of A260 nm. To be used as a template in SNP genotyping, DNA concentrations were equalized at concentrations of 4 ng/µL.

### SNP primer and probe design

The SNP target used in this study was the *ABCG2 rs2231142* genetic variant, with the following information (Table 1). The SNP reagents were ordered from Thermo Fisher Scientific (Assay ID: C__15854163_70, cat. no. 4362691) through PT Enigma Saintia Solusindo, Tangerang, in Banten province.

**Table 1:** SNP information

SNP ID	*rs2231142*
Gene	*ABCG2*
Location	Chr.4: 88131171 on GRCh38
SNP type	SNP type transversion substitution, missense mutation, intragenic
Context sequence	GCAAGCCGAAGAGCTGCTGAGAACT*[G/T]*TAAGTTTTCTCTCACCGTCAGAGG
Polymorphism	G/T

### SNP genotyping

SNP genotyping reaction mix is presented in Table 2. Polymerase chain reaction (PCR) amplification was carried out using the *QuantStudio 5* Real-Time PCR (*Applied Biosystems*) with the following three thermal cycles: (i) activation of enzymes for 20 s at 95 °C, (ii) DNA denaturation for 3 s at 95 °C, and (iii) annealing and extension for 30 s at 60 °C. The denaturation, annealing, and extension stages were carried out in 50 cycles.

**Table 2:** SNP genotyping reaction mix components

Components	Volume
TaqMan GTXpress Master Mix (2 ×)	5 µL
TaqMan SNP Genotyping Assay (20 ×)	0.5 µL
DNA sample	2.5 µL
Nuclease-free water (NFW)	2 µL
**Total volume per reaction**	10 µL

### Statistical analysis

All the data were rechecked to ensure its accuracy. The data were managed using Excel and analyzed using appropriate tools of Statistical Package for the Social Sciences (SPSS) software. Data were summarized and presented descriptively using tables. To compare data (anthropometric, clinical, and blood chemical) between the highland and lowland/coastal groups, we did a test of normality of the data distribution. Non-normally distributed data were transformed using Box-Cox transformation to find if the data could be normalized. Normally distributed data were analyzed using an independent *T*-test, while non-normally distributed data were analyzed using Mann–Whitney *U*-test. Correlation analysis was performed using Pearson correlation with a significance test using two-tailed for normally distributed data and passing the linearity test. Otherwise, the data were analyzed using Spearman-rank correlation. The Hardy–Weinberg equilibrium (HWE) was tested with the chi-square goodness-of-fit test. The *p* > 0.05 means the proportion of genotype frequencies according to the HWE principle. Also, the calculation of chi-square was carried out to see genotype and allele frequencies distribution between the highland and lowland groups.

## Results

### Tribes and languages of participants

Table 3 presents the distribution of participants according to region, tribe, and language. The highland Papuans are characterized by the linguistic group of the language they use. Their languages are Trans-New Guinea languages. On the other hand, lowland Papuans speak another family of Papuan languages or Austronesian. Our participants were not distributed equally in relation to location in Papua. The participants from lowland areas (78 participants) came from 12 regions and spoke 14 separated languages. They were predominantly Nafri, Tobati, and Kayo speakers from the Jayapura region (32 participants), Biak people (14 participants), Sentani people (9 participants), and Arfak (8 participants). The remaining tribal groups consisted of 1 to 3 participants. The highland group (65 participants) came from 15 regions and used 15 separated languages. They were mainly from the Dani (18 participants), Lani (15 participants), and Damal tribes (7 participants). The remaining groups consisted of not more than five participants.

**Table 3:** Participants distribution according to region, tribe, and language in West Papua

Region	Tribe/regional origin	Language		Frequency
		**Language used**^a^	**Linguistic group**^b^	***n* (%)**
Lowland/coastal	Sentani	Sentani	Another family of Papuan languages	9 (12%)
	Genyem	Mekwei		1 (1%)
	Tanah Merah	Tabla		3 (4%)
	Arfak	Meyah		8 (10%)
	Biak	Biak		14 (18%)
	Serui	Ambai		5 (6%)
	Ormu Wari	Ormu		1 (1%) 1 (1%) 32 (41%) 1 (1%)
	Yokari Jayapura Fak-fak	Yokari Nafri, Tobati, Kayo Iha		
	Moor	Moor		1 (1%)
	Sarmi	Isirawa		2 (3%)
Highland	Lani	Lani	Trans-New Guinea language	15 (23%)
	Dani	Dani		18 (28%)
	Amungme	Amungme		4 (6%)
	Damal	Damal		7 (11%)
	Ngalum	Ngalum Ok		5 (8%)
	Yali	Yali Pass Valley		2 (3%)
	Ketengban	Ketengban		1 (2%)
	Nduga	Nduga		4 (6%)
	Mee	Ekari Mee		3 (5%)
	Kamoro	Komoro		1 (2%)
	Moni	Moni		1 (2%)
	Mee & Amungme	Ekari, Mee, Amungme		4 (6%)
	Mee & Ngalum	Ekari, Mee, Ngalum		
	Amungme & Dani	Amungme, Dani		
	Marind & Dani	Marind, Dani		

^a^ [19], ^b^ [20]

### Anthropometric characteristics

We present anthropometric data of participants from the highlands and lowlands/coast and compare the mean values of the two groups, according to sex (Table 4). We conducted a normality test of the data distribution using the Shapiro–Wilk Test and found that some variables were not normally distributed. We further-up by carrying out statistical tests to compare the average values of measurements between the lowland and highland Papuans according to sex either using the *T*-test or Mann–Whitney *U*-test depending on whether the data is distributed normally or not (Table 4; see Additional file 1; see Additional file 2). We found that the two groups showed significant differences in their anthropometric profiles. The lowland Papuan men were lighter and taller than the highland Papuan men. Considering their common obesity, the men from the lowland were categorized on average as normal in contrast to those from the highland categorized as overweight (lowland: 23.5 ± 4.5; highland: 25.8 ± 3.17; *p* = 0.014), using Asian Standard. The same results were also shown by BMI in women (lowland: 24.3 ± 5.6; highland: 26.7 ± 4.0; *p* = 0.117). Anthropometric differences between the two groups were even more evident in the central obesity indicators (WC, WHR, WHtR), especially in women.

**Table 4:** Anthropometrics, clinical, and blood chemistry differences of West Papuan lowland and highland participants

Variables	Men mean ± SD						*p*	Women mean ± SD						*p*
	**Lowland (*n* = 45)**			**Highland (*n* = 40)**				**Lowland (*n* = 33)**			**Highland (*n* = 25)**			
Age (yr)	23.1	±	2.6	22.1	±	2.00	0.030*	22.3	±	2.2	20.7	±	2.6	0.007*
BW (kg)	62.3	±	11.9	66.2	±	8.22	0.124	59.1	±	15.2	62.8	±	10.3	0.380
BH (cm)	163.0	±	5.3	160.2	±	4.78	0.007	155.7	±	5.2	153.4	±	4.9	0.091
BMI (kg/m^2^)	23.5	±	4.5	25.8	±	3.17	0.014	24.3	±	5.6	26.7	±	4.0	0.117
WC (cm)	78.9	±	7.8	81.1	±	7.34	0.214	77.1	±	9.1	84.5	±	10.2	0.008
HC (cm)	92.4	±	8.5	92.2	±	6.77	0.992	92.5	±	10.6	98.2	±	8.4	0.044
WHR	0.85	±	0.05	0.88	±	0.04	0.072	0.84	±	0.06	0.88	±	0.05	0.017
WHtR	0.48	±	0.05	0.51	±	0.05	0.049	0.495	±	0.05	0.556	±	0.07	0.001
Biceps (mm)	3.5	±	2.1	3.4	±	1.82	0.971	5.4	±	2.0	7.7	±	2.9	0.001*
Triceps (mm)	3.9	±	2.1	5.1	±	2.13	0.002	6.2	±	2.0	12.4	±	5.1	0.000
Subscapular (mm)	8.3	±	3.7	7.5	±	3.70	0.175	10.7	±	3.3	18.2	±	6.9	0.000*
Suprailiac (mm)	10.0	±	3.5	10.3	±	3.71	0.829	11.6	±	4.5	22.8	±	7.9	0.000*
BF (%)	10,2	±	4.5	10.6	±	4.04	0.539*	13.9	±	3.8	28.7	±	4.0	0.000
TF (kg)	6.7	±	3.8	7.1	±	3.29	0.460*	8.4	±	3.9	18.3	±	5.0	0.000
UA (mg/dL)	5.8	±	1.9	6.6	±	1.45	0.097	5.9	±	1.5	6.9	±	2.2	0.041
TC (mg/dL)	157.5	±	35.6	159.7	±	38.84	0.767	145.6	±	40.6	167.1	±	45.1	0.007
FBG (mg/dL)	86.5	±	11.8	79.9	±	11.24	0.019	88.7	±	9.8	75.8	±	10.5	0.000
RBG (mg/dL)	104.5	±	20.7	101.8	±	18.08	0.484	101.5	±	18.2	101.2	±	17.4	0.955
SBP (mmHg)	122.9	±	8.2	122.4	±	11.04	0.976*	113.6	±	9.7	108.2	±	8.0	0.031
DBP (mmHg)	81.9	±	7.3	80.2	±	7.84	0.419*	79.2	±	7.8	74.6	±	8.4	0.052*

*T*-test, otherwise with Mann–Whitney *U*-test*

#### Body fat (BF)

Table 4 presents the results of measurements of subcutaneous fat thickness at four locations (biceps, triceps, subscapular, and suprailiac). It shows that the highlander women have much thicker subcutaneous fat compared to those from the lowlands/coast, with highly significant statistical differences. In men, both groups showed more similarities, except for triceps where men from the highland had thicker triceps (*p* = 0.002).

### Clinical characteristics

We compared blood chemicals concentration and blood pressure between lowland/coastal Papuans and highland Papuans (Table 4). A higher concentration of UA and total cholesterol were found in highland men and women, with a more significant difference in uric acid levels in men and total cholesterol levels in women. This condition is in contrast to fasting glucose levels in both sexes, where participants from lowlands/coastal areas have higher levels compared to those from highlands, with a very significant statistical difference. Interestingly, random blood sugar levels did not behave the same as fasting blood sugar, where both groups of participants showed no difference in mean values. For blood pressure, the SBP and DBP values are consistently higher than those from lowland/coast, but a statistical difference is detected only in women.

### Correlation between anthropometrics and clinical variables

We have witnessed striking differences in anthropometric, blood chemistry, and clinical profiles between participants from the Papuan highlands and those from the lowlands/coast and sex. We wanted to know how these variables, especially anthropometric and clinical variables, were related. Therefore, we conducted correlation analysis according to region of origin and sex. In choosing the correct correlation analysis, we did normality and linearity tests for correlation analysis (see Additional file 3). We discovered that the UA concentration and DBP were significantly correlated with obesity in lowland/coastal Papuans, as clearly seen in their correlations with general obesity and central obesity variables. This is in contrast to the situation in women from the highland. In this group, UA tends to show an opposite relation with obesity (Table 5). In men from the lowland/coast, the UA correlate significantly with BW but not with other obesity indicators. Regarding the relation between DBP and obesity, significant correlation was shown by common obesity (BW, BMI) and some regional obesity indicators (WC, WHtR) in women and men from lowland/coast but not for those from the highland.

**Table 5:** Correlation analysis between clinical and blood chemistry with anthropometric differences of West Papuan lowland and highland students

**Sex**	**Clinical variables**	**Location** **(L/H)**	**Anthropometric variables**										
			**Age**	**BW**	**BH**	**BMI**	**WC**	**HC**	**WHR**	**WHtR**	**Triceps**	**BF**	**Total fat**
Men	UA	L	0.12	0.358*	0.14	0.00	0.00	0.00	0.00	0.00	0.00	0.19	0.00
		H	− 0.16	0.12	0.03	0.07	0.11	0.09	0.09	0.10	− 0.06	0.08	0.10
	TC	L	0.12	0.00	− 0.06	0.00	0.00	0.00	0.00	0.00	0.00	0.12	0.10
		H	− 0.363*	0.23	− 0.05	0.330*	0.19	0.20	0.06	0.19	0.08	0.26	0.29
	FBG	L	0.18	0.28	0.15	0.00	0.00	0.00	0.00	0.00	0.00	0.17	0.25
		H	− 0.04	0.12	0.17	0.26	− 0.04	0.18	− 0.39	− 0.09	− 0.28	− 0.35	− 0.28
	RBG	L	− 0.06	0.12	− 0.02	0.00	0.00	0.00	0.00	0.00	0.00	− 0.07	0.01
		H	− 0.26	− 0.10	0.16	− 0.21	0.05	− 0.09	0.27	− 0.01	0.16	0.17	0.09
	SBP	L	− 0.08	0.10	− 0.04	0.00	0.20	0.15	0.10	0.00	0.23	0.24	0.21
		H	− 0.03	0.08	− 0.47	0.364*	0.13	0.17	− 0.03	0.27	0.26	0.336*	0.316*
	DBP	L	0.13	0.451**	0.09	0.00	0.418**	0.426**	0.03	0.00	0.23	0.294*	0.389**
		H	0.01	− 0.03	− 0.44	0.12	− 0.03	− 0.05	0.02	0.11	0.07	0.23	0.20
Women	UA	L	0.09	0.415*	− 0.05	0.471**	0.28	0.413*	− 0.20	0.32	0.364*	0.33	0.433*
		H	0.21	− 0.15	0.11	− 0.22	− 0.28	− 0.13	− 0.40*	− 0.31	− 0.31	− 0.36*	− 0.27
	TC	L	0.17	0.22	− 0.02	0.26	0.33	0.19	0.07	0.353*	0.11	− 0.16	− 0.05
		H	0.32	0.37	0.25	0.29	0.23	0.24	0.20	0.17	0.04	− 0.04	0.18
	FBG	L	− 0.07	− 0.19	− 0.31	− 0.09	− 0.34	− 0.19	− 0.27	− 0.28	0.06	0.14	0.05
		H	0.14	− 0.11	− 0.02	− 0.11	− 0.21	0.00	− 0.42*	− 0.20	− 0.21	− 0.26	− 0.20
	RBG	L	0.12	0.15	0.08	0.17	0.28	0.07	0.376*	0.29	− 0.21	− 0.28	0.00
		H	− 0.06	− 0.04	0.15	− 0.10	0.05	− 0.06	0.18	− 0.01	0.05	− 0.11	− 0.09
	SBP	L	− 0.05	0.20	0.17	0.07	0.19	0.23	− 0.02	0.20	− 0.13	− 0.28	− 0.16
		H	− 0.32	0.27	0.13	0.13	0.12	0.26	− 0.10	0.07	0.01	− 0.16	0.03
	DBP	L	0.04	0.32	0.11	0.361*	0.355*	0.33	− 0.09	0.349*	0.07	0.06	0.16
		H	− 0.27	0.11	− 0.03	0.13	0.13	0.25	− 0.08	0.13	0.01	− 0.03	0.07

*L*, lowland; *H*, highland; * or ** indicating significance of correlation

### ABCG2 rs2231142 genotype and allele frequency

Table 6 presents *ABCG2 rs2231142* genotype and allele frequencies of highland and lowland/coastal Papuans with the HWE statistical tests. It shows that the *χ*^2^ value for the lowland/coastal Papuans (1.283) and the highland Papuans (0.366) is lower than *χ*^2^ Table (3.841) with a *p*-value higher than *α* = 0.05. This means the tests are not able to disprove that the genotypic and allele frequencies are in equilibrium.

**Table 6:** *ABCG2 rs2231142* genotype and allele frequencies of highland and lowland/coastal Papuan

Regional origin	*n*	Genotype, *n* (%)			HWE		Allele, *n* (%)	
		**GG**	**GT**	**TT**	***χ***^**2**^	p*	**G**	**T**
Lowland/coastal Papuan	78	46 (59.0)	30 (38.5)	2 (2.6)	1.283	0.257	122 (78.2)	34 (21.8)
Highland Papuan	64	55 (85.9)	9 (14.1)	0 (0.0)	0.366	0.545	119 (93.0)	9 (7.0)
Total	142	101 (71.1)	39 (27.5)	2 (1.4)	0.672	0.412	239 (84.8)	43 (15.2)

^*^The *p* > 0.05 means the proportion of genotype frequencies meet the HWE law

Table 7 presents the genotype frequency, allele frequency, and odds ratio (OR) of the *ABCG2 rs2231142* variant of the lowland/coastal Papuans and highland Papuans according to sex. We found that the TT genotype was not found in highland and lowland/coastal Papuan men. Interestingly, we detected the presence of TT homozygotes in lowland/coastal female samples in small frequency (6.1%) or 2.6% of the total lowland/coastal participants. The GT heterozygote was much higher among lowland/coastal women (45.4%) than lowland/coastal men (33%), and this is much higher when compared to the heterozygote among highland women (8.3%) and highland men (17.9%). The *ABCG2 rs2231142* > T allele in lowland/coast far exceeds that in highland (21.8% vs 7.1%), both in men (16.7% vs 9.0%) and women (28.8% vs 4.2%), respectively.

**Table 7:** Genotype frequency, allele frequency, and odds ratio (OR) of *ABCG2 rs2231142* for Papuan participants according to sex

Sex	Genotype/allele	Lowland	Highland	*χ*^2^	*p*	OR	95% *CI*
**Genotype**							
Men	GG	30 (66.7)	33 (82.5)	2.767	0.096	2.357	0.846–6.565
	GT	15 (33.3)	7 (17.5)				
	TT	0 (0.0)	0 (0.0)				
Women	GG	16 (48.5)	22 (91.7)	11.761	0.003	11.688	2.359–57.914
	GT	15 (45.4)	2 (8.3)				
	TT	2 (6.1)	0 (0.0)				
All	GG	46 (59.0)	55 (85.9)	12.854	0.002	4.251	1.841–9.816
	GT	30 (38.5)	9 (14.1)				
	TT	2 (2.6)	0 (0.0)				
**Allele**							
Men	G	75 (83.3)	73 (91.2)	2.356	0.125	2.086	0.804–5.411
	T	15 (16.7)	7 (8.8)				
Women	G	47 (71.2)	46 (95.8)	11.210	0.001	9.298	2.049–42.201
	T	19 (28.8)	2 (4.2)				
All	G	122 (78.2)	119 (93.0)	11.928	0.001	3.685	1.694–8.014
	T	34 (21.8)	9 (7.0)				

In calculating the odd ratio (*OR*) value, the noncarrier genotype (*GG*) is a reference to the carrier genotype (GT + TT)

### Regional distribution of ABCG2 rs2231142 genotype according to original homeland in West Papua

**Fig. 1:**
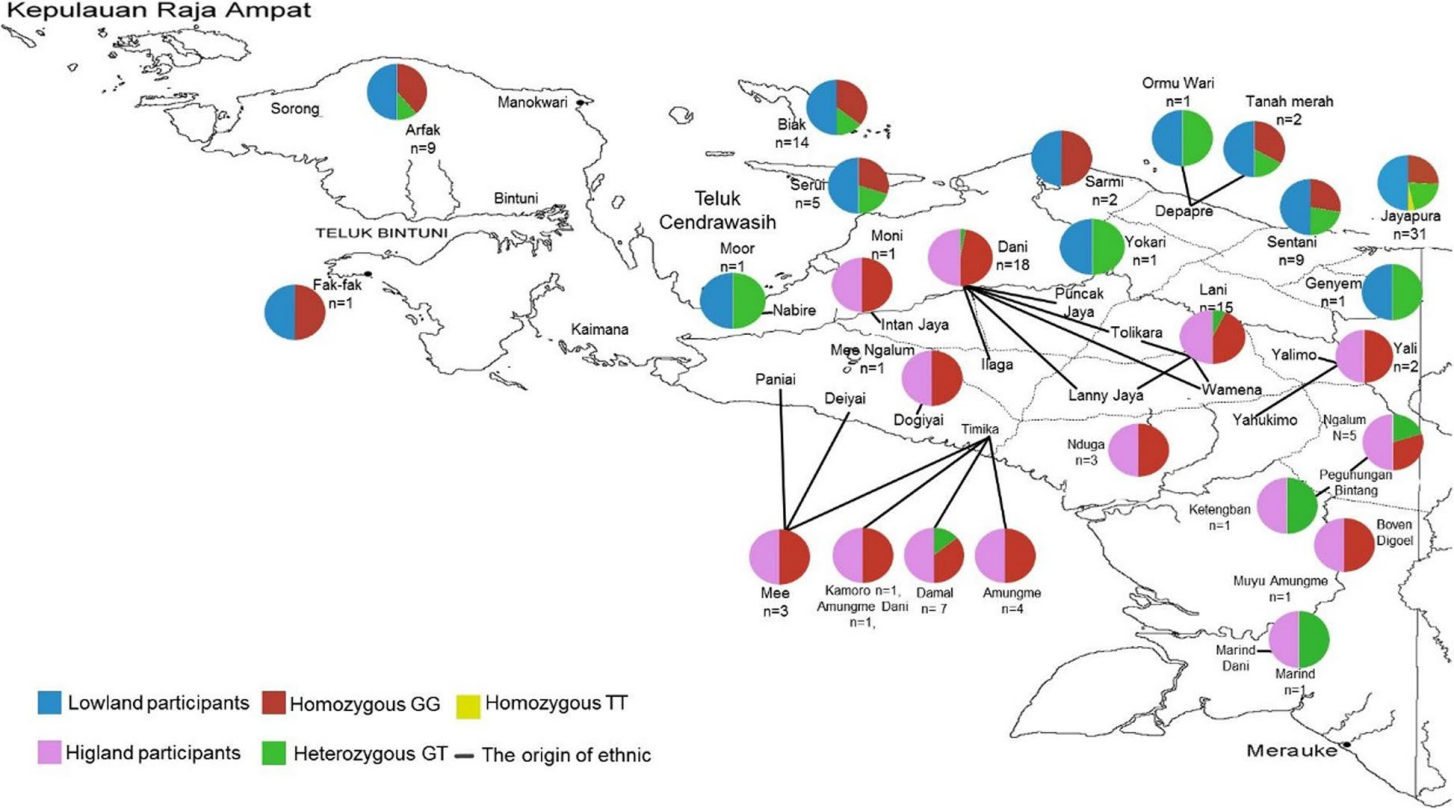
Distribution of *ABCG2 rs2231142* genotype (GG, GT, and TT) according to participants’ homeland. The background map is a copyright property of Trek-Papua [21]

**Fig. 2:**
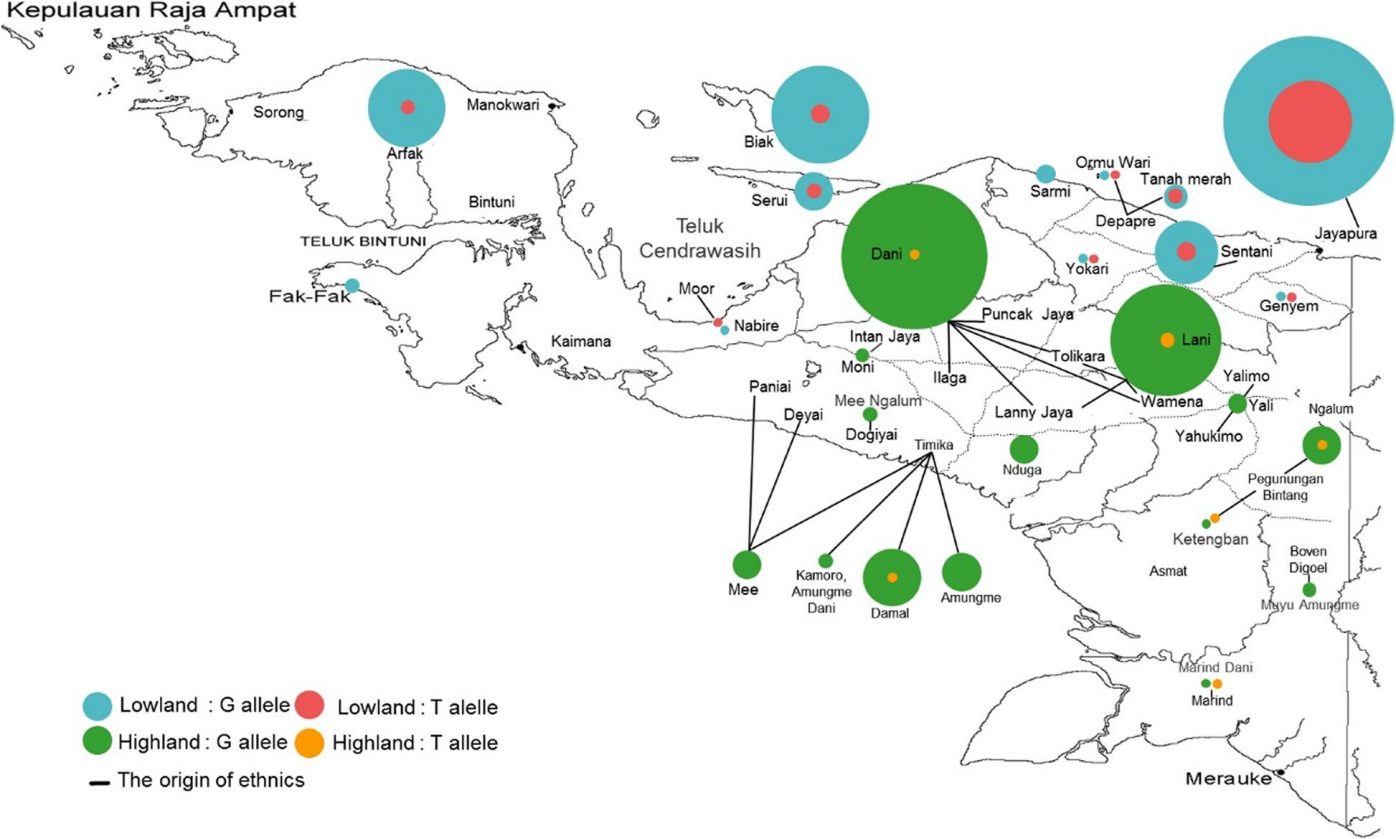
Distribution of *ABCG2 rs2231142* alleles G and T according to participants tribe origin. The background map is the copyright property of Trek-Papua [21]

We mapped the regional distribution of genotype according to participants’ homeland (Fig. 1). As almost all our participants were either homozygous GG or heterozygous GT, the only region in which the homozygous TT was present was the Jayapura region, a lowland area and the capital of the Papuan province (two participants). The map also shows that the heterozygous GT is present in almost all ethnic groups in lowland/coastal areas, except for Fakfak and Sarmi, indicating that the T allele penetrated lowland populations evenly. We also mapped the distribution of G or T alleles based on ethnicity according to the origin of the participants, both in lowland/coastal and highland areas (Fig. 2). The data shows that the alleles were distributed almost evenly across all regions, both in the lowland/coastal and highland samples.

### Bivariate analysis of the relationship between genotypes and measured phenotypes

We did a statistical comparison of genotypes (GG and GT) with anthropometric variables (BMI, WHR, and total BF) (see Additional file 4) and blood chemistry (see Additional file 5), in order to find possible value differences among anthropometric variables and clinical variables with genotypes. The data shows that there were no significant differences between both anthropometric and clinical values with their genetic composition (GG or GT) for either men or women. Interestingly, statistical tests indicated that women’s fasting blood sugar differed significantly between the GG and GT genotypes (*GG*: 84.7 ± 9.1 mg/dL; *p* = 0.040) (see Additional file 5).

## Discussion

The research findings highlight five key issues that required attention: participant representation, differences in anthropometric characteristics, clinical chemistry, metabolic health and adaptation of Papuans, and the distribution of alleles within the population.

### Participants’ representation from the lowlands and highlands of Papua

Our research on indigenous Papuan students at Satya Wacana University began with the observation that Papuan students have been studying there since the 1970s, and their numbers have grown over the years, reflecting increasing ethnic and regional diversity. Before West Papua was divided into two provinces (and now becoming six provinces), most Papuan students at the university came from the lowland areas. Those from the highlands, of whom there were only a few, were mostly from migrant families from the lowland/coastal areas, particularly from the Biak and Serui tribes or from Jayapura and the surrounding areas. At that time, they went to the interior to work mostly as teachers or religious workers. The regional expansion and autonomy program (Program Pemekaran Wilayah dan Otonomi Daerah), which grants more authority to local governors and district heads, including the initiative to send high school graduates to universities outside Papua, has significantly increased the number of Papuan students in universities across Indonesia. This includes students from the highlands, with Satya Wacana in Salatiga, Central Java, being one of the institutions that has seen a rise in Papuan enrollment. By the time this research was conducted, at least 770 students from West Papua were enrolled at Satya Wacana, not counting those who were inactive or not registered in 2018. Although our sample did not represent every tribe from the highlands or lowlands/coastal regions, it included the largest tribes from both areas: Dani, Lani, Yali, Mee, and Amungme from the highlands and Biak, Serui, Sentani, Sarmi, Arfak, Fakfak, and tribes around Jayapura from the coast and lowlands.

### Anthropometric features of the highland and lowland/coastal participants

Our anthropometric data reveals striking differences among Papuans based on sex and eco-regions in terms of height, whole-body fat distribution, and regional fat distribution. In relation to stature, both men and women, the lowland/coastal Papuan is taller than the highland Papuan. This difference may be partly explained by their genetic composition, and that the lowland/coastal Papuan is genomically a mix of the Australoid- and the Austronesian-speaking Mongoloids, creating a distinct Melanesian genetic composition predominantly in the lowlands/coast [22], with a taller stature [23] compared to the Papuan mountains population [24]. Functional studies have attempted to explain that the shorter stature of the highland Papuan is not due to growth hormone problems or protein status [25]. It could be that shorter stature is an adaptation mechanism to poor and changing conditions of nutrient availability in the past [26] or to balance other energetic needs. This trait appears to be responsive to ecological changes, modernity, disease epidemiology, and improvements in socioeconomic conditions [27]. Interestingly, obesity was significantly higher in those from highland than in those from lowland/coast, as indicated by BW and BMI, despite shorter stature. The difference was mainly explained by increased fatness (as indicated by triceps skinfold thickness) and higher central adiposity in those from highland (as shown by WHR and WHtR) in men but more so in women. Fat deposits under the skin make a significant contribution, as pronounced strongly in women. Higher fat deposit in those from highland may be an adaptation to the lower temperatures in the region that favor greater peripheral energy storage [28, 29]. This implies also that increased obesity in Papuan students from highland indicates that they are more sensitive to the obesogenic and modern environment of Salatiga but also indicating higher storage flexibility and capacity. The less pronounced differences in BW and BMI between highland and lowland groups in our study, especially women, may be explained by individual variability, the socio-economic conditions of the students, and the obesogenic/modern environment of Salatiga city. Moreover, better financial support provided by Papuan district government scholarships compared to 20 years ago may have moderated differences in weight and obesity among these groups.

### Metabolic health and adaptation of highland Papuan

Our anthropometric and blood chemistry data reveal distinct differences between highland and lowland/coastal Papuans. Highland Papuans exhibited higher concentrations of uric acid (UA) and total cholesterol (TC) but lower fasting blood glucose (FBG) and blood pressure levels compared to their lowland/coastal counterparts (Table 4). These elevated UA and TC levels align with the higher levels of obesity and adiposity observed among highland participants, a pattern reported in previous studies, including those involving youth [30].

Given the established link between hyperuricemia and metabolic disorders such as insulin resistance, metabolic syndrome, and type 2 diabetes mellitus (T2DM) [31], we conducted a correlation analysis by region and sex. Surprisingly, among highland Papuan women, UA levels were negatively correlated with several obesity indicators, including a significant negative correlation with waist-to-hip ratio (WHR) and a weaker one with body fat percentage (BF%) (Table 1). WHR also showed strong associations with other markers such as body weight (BW), body mass index (BMI), waist circumference (WC), waist-to-height ratio (WHtR), triceps skinfold, and BF% (*p* < 0.01). These findings suggest that elevated UA in highland Papuans may not pose the typical metabolic risk associated with obesity. Moreover, all obesity indicators, including BF%, were inversely associated with FBG, with a strong negative correlation between WHR and FBG (*r* = − 0.42*), indicating a potential adaptive mechanism. We propose that highland Papuans may exhibit greater adipose tissue plasticity, allowing fat storage without metabolic dysfunction, possibly as a physiological response to the colder temperatures of high-altitude environments. The increased UA levels may reflect enhanced metabolic production or increased renal reabsorption of UA as part of this adaptation. The high UA levels in highland Papuans could function as a first-line antioxidant, neuroprotector, and immune-protector [32], which increases its production under hypoxic conditions at high altitude [33], particularly in the nasal cavity area [34] as well as in the upper and lower respiratory tract and lung epithelial lining fluids [35].

Regarding to a lower FBG in Highlander Papuans, the finding is supported by other study [36]. We thought that it is part of long-term adaptation to the region’s low-temperature potentially involving non-shivering thermogenic metabolism in BAT [37] or coordinated thermogenic activity with BAT [38], which promotes glucose uptake in adipocytes and contributes to a significant reduction in blood glucose levels. Another alternative is the involvement of UCP1-independent thermogenesis mediated by beige fat which functions as a “glucose sink” that improves glucose tolerance independent of body weight loss [39–42].

In relation to the lower blood pressure of participants from highland, which was particularly evident in women, it is worth noting because it is associated with much higher central obesity and higher regional fat distribution. There have been reports indicating that BAT can lower blood pressure, with these effects being more pronounced at higher BMIs [37]. It is possible that in highland Papuans, low-temperature adaptation combined with reduced ambient oxygen has recruited mechanisms promoting adipogenesis, angiogenesis, and neurite outgrowth as part of their adaptation to low atmospheric pressure and oxygen concentration, in addition to thermoregulation across adipose tissues [43] as well as increased efficiency of O_2_ transfer and utilization to maintain metabolic and physiologic homeostasis [44].

Our results, however, raise a concern. Clinical studies have shown that serum UA is associated significantly with BAT in humans and can inhibit BAT thermogenic capacity [45]. HUA also has comorbidity with several metabolic diseases, primarily due to adipose tissue dysfunction [46]. The question is to what extent the increasing obesity, particularly central obesity, can be tolerated at higher UA concentrations without impairing adipose tissue function in highland Papuans. Since our data comes from young adults, it would be interesting to explore metabolic homeostasis in older highland Papuans.

### Metabolic health of lowland Papuan

Clinical data for lowland/coastal Papuans reveal a different profile compared to the highland Papuans. The most notable difference is that the fasting blood sugar levels of lowland/coastal Papuans are significantly higher than those of highland Papuans. The distribution of our sample from a variety of locations in lowland/coastal regions suggests that this characteristic is related to variables prevalent across the region. To see how sensitive these traits are to new environmental exposures in Salatiga, we compared the differences in mean anthropometric and blood chemistry values of our participants based on the duration of exposure, namely < 3 years and ≥ 3 years (see Additional file 6). We found higher UA levels (6.5 ± 2 mg/dl vs 4.8 ± 0.8 mg/dl; *p* = 0.386) and fasting blood sugar levels (87.1 ± 13.3 vs 85.3 ± 8.3 mg/dl; *p* = 0.11) in those who have been exposed to ≥ 3 years in Salatiga, especially in men, although statistically not significant. This clinical profile resembles the so-called Asian phenotype, in which metabolic derangements occur at a lower BMI. For example, East Asians develop T2DM at a higher rate, at a younger age, and with a lower BMI than their European counterparts [47]. T2DM among Asians tends to be more closely associated with the insulin-sensitivity phenotype, suggesting that the Asian phenotype is related to the lower storage capacity and plasticity of the fat tissue [48]. Our interpretation of the higher FBG levels in lowland/coastal Papuans and the potential link to the Asian phenotype is supported by archeological [49] and genetic studies [8, 11, 14, 50] which indicate genetic admixture in lowland/coastal Papuans following the arrival of Austronesian speakers and other group from mainland Asia around 3000 years or so. It would be valuable to investigate this phenomenon further by comparing the FBG levels of lowland/coastal and highland Papuans in older age groups.

### Genotype and allele frequency of ABCG2 rs2231142 in lowland and highland Papuans

Our research found that the genotype and allele frequency of *ABCG2 rs2231142* > T differ significantly between those from highland Papua and those from the lowland/coastal areas. Those from the lowland/coastal areas exhibit a much higher frequency of the minor allele than those from the highlands, a pattern observed in both men and women. Women from the highland regions have the lowest frequency of the minor allele, which contrasts sharply with its presence in women from the lowland/coastal regions. The homozygote TT genotype is completely absent in both men and women from the highlands, suggesting that the minor allele of *ABCG2 rs2231142* > T is not carried by the native population. It appears that the allele originated from Neolithic migrants which arrived from Southeast Asia during the Late Holocene period (approximately 3200 years ago) was admixed into the existing native population, and became fixed in the population, as indicated by the HWE statistical tests, which show that the genotypic and allele frequencies are in equilibrium (Table 6) The presence of Wañelek pottery in highland Papua, along with the expansion of Lapita pottery in the Western Pacific, supports this hypothesis [51]. Additionally, the very low frequency of the *ABCG2 rs2231142* > *T* variant in African Americans (3%) and Africans (2.8%) [52] supports our finding. In contrast, the *ABCG2 rs2231142* > *T* has a high frequency in East Asian and Southeast Asian populations, culminating in the Philippine population by 46% [53] and some tribes in Indonesia (Karwur, unpublished). The *ABCG2 rs2231132* > T minor allele frequency tends to be lower when it is away from East Asia and Southeast Asia. In Oceania, the frequency of the minor allele ranges between 12.7% for Native Hawaiians and 31.1% for Samoans [54]. Its presence in the highland Papuans is the lowest among the Oceanian.

### Weakness of the research

This research was conducted with West Papuan students in Salatiga (± 650 m above sea level), with an average age of 21–23 years, who had been living in Salatiga for at least 6 months. Salatiga, to some extent, has a more obesogenic environment compared to their native land. More specific studies are needed to compare particular tribes from the Central Mountains of West Papua with those from the lowland/coastal areas, using larger participants with comprehensive clinical and metabolic indicators.

## Conclusions

This study demonstrated significant differences in anthropometric measurements, BF, and blood chemistry between students from highland and lowland/coastal areas. While individuals from highland areas exhibited high adiposity and subcutaneous fat folds, these were not accompanied by blood chemistry and clinical disorders typically observed in Asian populations. In contrast, individuals from lowland/coastal West Papua, despite having lower levels of obesity, showed elevated FBG, UA, and DBP, alongside increased general obesity and adiposity. This condition appears to be mitigated by fat distribution in the lower body among individuals from both highland and lowland/coastal areas. The observed differences between these populations suggest distinct local adaptations to their specific environmental conditions and genetic backgrounds. Notably, the high frequency of the genetic variant *ABCG2 rs2231142* > T allele in lowland/coastal populations supports the hypothesis of ISEA as the origin of this allele. Further research is needed to identify physiological and molecular components, as well as mechanistic explanations for the metabolic differences observed between these populations, such as variations in carbohydrate-lipid metabolism, fat partitioning among storage organs, and insulin signaling and sensitivity. Our findings highlight the potential for differences in treatment responses between highland and lowland populations due to their unique environmental contexts, including differences in food and nutritional environments. Comprehensive and longitudinal studies are urgently required to further explore these findings.

## Supplementary Information

Additional file 1. Tests of Normality Shapiro–Wilk Before and After Data Transform & Mean Differences Tests: Highland and Lowland/Coast Men.

Additional file 2. Tests of Normality Shapiro–Wilk Before and After Data Transform tests & Mean Differences Tests: Highland and Lowland/Coast Women.

Additional file 3. Summary Tables of Normality and Linearity Tests for Correlation Analysis Between Clinical and Anthropometric Data of Lowland Highland Men.

Additional file 4. Comparison of *ABCG2 rs22231142* Variant Against Anthropometry of Papuan Participants in Salatiga.

Additional file 5. Comparison of *ABCG2* rs22231142 Variant Against Blood Chemistry and Clinical Data of Papuan Participants in Salatiga.

Additional file 6. Anthropometric and blood chemistry values of Papuan students from lowland and highland areas based on length of stay in Salatiga (average ± SD).

## Data Availability

The data sets used and/or analyzed during the current study are available from the corresponding author on reasonable request.

## Abbreviations

ABCG2: ATP-binding cassette subfamily G member 2
ABCC11: ATP-binding cassette subfamily C member 11
ADP: Adenosine diphosphate
ATP: Adenosine triphosphate
BAT: Brown adipose tissue
BF: Body fat
BH: Body height
BMI: Body mass index
BW: Body weight
CI: Confidence interval
DBP: Diastolic blood pressure
DNA: Deoxyribonucleic acid
FBG: Fasting blood glucose
GDP: Guanosine diphosphate
GTP: Guanosine triphosphate
HWE: Hardy-Weinberg equilibrium
HC: Hip circumference
ISEA: Island South East Asia
NFW: Nuclease-free water
MAF: Minor allele frequency
OR: Odds ratio
PCR: Polymerase chain reaction
PNG: Papua New Guinea
RBG: Random blood glucose
RyR2: Ryanodine receptor 2
SERCA: Sarco Endoplasmic Reticulum Ca^2+^-ATPase
SBP: Systolic blood pressure
SNP: Single-nucleotide polymorphism
SPSS: Statistical Package for the Social Sciences
TC: Total cholesterol
T2DM: Type-2 diabetes mellitus
UA: Uric acid
UCP1: Uncoupling protein 1
UV: Ultraviolet
UV-VIS: Ultraviolet-visible spectrophotometer
WAT: White adipose tissue
WC: Waist circumference
WHR: Waist-to-hip ratio
WHtR: Waist-to-height ratio

## References

[1] Blüher M. Obesity: global epidemiology and pathogenesis. Nat Rev Endocrinol. 2019;15(5):288–8. 10.1038/s41574-019-0176-8 30814686

[2] Roth GA, Forouzanfar MH, Moran AE, Barber R, Nguyen G, Feigin VL, et al. Demographic and epidemiologic drivers of global cardiovascular mortality. N Engl J Med. 2015;372(14):1333–41. 25830423 10.1056/NEJMoa1406656 PMC4482354

[3] Stuckler D, McKee M, Ebrahim S, Basu S. Manufacturing epidemics: the role of global producers in increased consumption of unhealthy commodities including processed foods, alcohol, and tobacco. PLoS Med. 2012;9(6):e1001235. 10.1371/journal.pmed.1001235 PMC3383750 22745605

[4] Paul BJ, James R. Gout: an Asia-Pacific update. Int J Rheum Dis. 2017;20(4):407–16. 10.1111/1756-185X.13103 28585370

[5] Abarca-Gómez L, Abdeen ZA, Hamid ZA, Abu-Rmeileh NM, Acosta-Cazares B, Acuin C, et al. Worldwide trends in body-mass index, underweight, overweight, and obesity from 1975 to 2016. The Lancet. 2017;390:2627–42. 10.1016/S0140-6736(17)32129-3 PMC5735219 29029897

[6] Johnson RJ, Lanaspa MA, Sanchez-Lozada LG, Rivard CJ, Bjornstad PS, Merriman T, et al. Fat storage syndrome in Pacific peoples: a combination of environment and genetics?. Pac Health Dialog. 2014;20(1):11–6. 25928990

[7] White JP. In The Prehistory of Polynesia . Harvard University Press; 1979. 352–377 p.

[8] Pedro N, Brucato N, Fernandes V, André M, Saag L, Pomat W, et al. Papuan mitochondrial genomes and the settlement of Sahul. J Hum Genet. 2020;65(10):875–87. 10.1038/s10038-020-0781-3 PMC7449881 32483274

[9] Lum JK, Jorde LB, Schiefenhovel W. Affinities among Melanesians, Micronesians, and Polynesians: a neutral, biparental genetic perspective. Hum Biol. 2002;74(3):413–30. 10.1353/hub.2002.0031 12180764

[10] André M, Brucato N, Hudjasov G, Pankratov V, Yermakovich D, Montinaro F, et al. Positive selection in the genomes of two Papua New Guinean populations at distinct altitude levels. Nat Commun. 2024;15(1):3352. 38688933 10.1038/s41467-024-47735-1 PMC11061283

[11] Xu S, Pugach I, Stoneking M, Kayser M, Jin L. Genetic dating indicates that the Asian-Papuan admixture through Eastern Indonesia corresponds to the Austronesian expansion. Proc Natl Acad Sci. 2012;109(12):4574–9. 10.1073/pnas.1118892109 PMC3311340 22396590

[12] Oliveira S, Nägele K, Carlhoff S, Pugach I, Koesbardiati T, Hübner A, et al. Ancient genomes from the last three millennia support multiple human dispersals into Wallacea. Nat Ecol Evol. 2022;(7):1024–34. 10.1038/s41559-022-01775-2 PMC9262713 35681000

[13] Bellwood P. A hypothesis for Austronesian origins. Asian Perspect. 1984;26(1):107–17.

[14] Redd AJ, Stoneking M. Peopling of Sahul: mtDNA variation in Aboriginal Australian and Papua New Guinean populations. Am J Hum Genet. 1999;65(3):808–28. 10.1086/302533 PMC1377989 10441589

[15] Jacobs GS, Hudjashov G, Saag L, Kusuma P, Darusallam CC, Lawson DJ, et al. Multiple deeply divergent Denisovan ancestries in Papuans. Cell. 2019;177(4):1010–21. 10.1016/j.cell.2019.02.035 30981557

[16] Okada Y, Sim X, Go MJ, Wu JY, Gu D, Takeuchi F, et al. Meta-analysis identifies multiple loci associated with kidney function-related traits in East Asian populations. Nat Genet. 2012;44(8):904–9. 10.1038/ng.2352 PMC4737645 22797727

[17] He W, Phipps-Green A, Stamp LK, Merriman TR, Dalbeth N. Population-specific association between ABCG2 variants and tophaceous disease in people with gout. Arthritis Res Ther. 2017;19:1–6. 10.1186/s13075-017-1254-8 PMC5341474 28270222

[18] World Health Organization. The Asia-Pacific perspective : redefining obesity and its treatment. Sydney: Health Communications Australia Pty Limited on behalf of the Steering Committee; 2000.

[19] Badan Pengembangan dan Pembinaan Bahasa. Kementerian Pendidikan dan Kebudayaan. 2019. Available from: http://118.98.223.79/petabahasa/provinsi.php?idp=Papua. Cited 2024 Apr 5.

[20] Ross M. Pronouns as a preliminary diagnostic for grouping Papuan languages. Canberra: Pacific Linguistics; 2005. 15–66 p. Available from: https://en.wikipedia.org/wiki/Trans-Fly%E2%80%93Bulaka_River_languages. Cited 2024 Apr 5.

[21] Trek Papua. Papua, Korowai tribe. 2018. Available from: https://trek-papua.com/baliem-valley-papua/korowai-batu-expedition/2-weeks-trekking-to-the-south-east/.

[22] Bergström A, Oppenheimer SJ, Mentzer AJ, Auckland K, Robson K, Attenborough R, et al. A Neolithic expansion, but strong genetic structure, in the independent history of New Guinea. Sci. 2017;357(6356):1160–3. 10.1126/science.aan3842 PMC5802383 28912245

[23] Pietrusewsky M. Lapita-associated skeletons from Watom Island, Papua New Guinea, and the origins of the Polynesians. Asian Perspect. 1988;28(1):83–9. Available from: https://scholarspace.manoa.hawaii.edu/server/api/core/bitstreams/32232089-9254-4c5d-b2d9-59b8fd8d4a23/content . Cited 2025 Apr 17.

[24] Rikimaru T, Fujita Y, Okuda T, Kajiwara N, Date C, Heywood PF, et al. Utilization of urea nitrogen in Papua New Guinea highlanders. J Nutr Sci Vitaminol. 1985;31(3):393–402. Available from: https://www.jstage.jst.go.jp/article/jnsv1973/31/3/31_3_393/_pdf/-char/en . Cited 2025 Apr 4. 10.3177/jnsv.31.393 4067671

[25] Schwartz J, Brumbaugh RC, Chiu M. Short stature, growth hormone, insulin-like growth factors, and serum proteins in the Mountain Ok people of Papua New Guinea. J Clin Endocrinol Metab. 1987;65(5):901–5. 10.1210/jcem-65-5-901 3667885

[26] Hyndman DC, Ulijaszek SJ, Lourie JA. Variability in body physique, ecology, and subsistence in the Fly River region of Papua New Guinea. Am J Phys Anthropol. 1989;79(1):89–101. 10.1002/ajpa.1330790110 2750882

[27] Ulijaszek SJ. Socio-economic factors associated with physique of adults of the Purari Delta of the Gulf Province, Papua New Guinea. Ann Hum Biol. 2003;30(3):316–28. 10.1080/0301446031000086004 12850964

[28] Wells JCK. Ecogeographical associations between climate and human body composition: analyses based on anthropometry and skinfolds. Am J Phys Anthropol. 2012;147(2):169–86. 10.1002/ajpa.21591 22212891

[29] Isshiki M, Naka I, Kimura R, Nishida N, Furusawa T, Natsuhara K, et al. Admixture with indigenous people helps local adaptation: admixture-enabled selection in Polynesians. BMC Ecol Evol. 2021;21:1–3. 10.1186/s12862-021-01900-y PMC8456657 34551727

[30] Niu Y, Zhang Y, Sun Y, Sheng J, Lu W, Li J, et al. A combined association of obesity, alanine aminotransferase and creatinine with hyperuricemia in youth aged 13–20 years. Front Nutr. 2024;20:11. 10.3389/fnut.2024.1326039 PMC11223030 38966416

[31] Du L, Zong Y, Li H, Wang Q, Xie L, Yang B, et al. Hyperuricemia and its related diseases: mechanisms and advances in therapy. Signal Transduct Target Ther. 2024;9(1):212. 39191722 10.1038/s41392-024-01916-y PMC11350024

[32] Ames BN, Cathcart R, Schwiers E, Hochstein P. Uric acid provides an antioxidant defense in humans against oxidant- and radical-caused aging and cancer: a hypothesis. Proc Natl Acad Sci. 1981;78(11):6858–62. 10.1073/pnas.78.11.6858 PMC349151 6947260

[33] Vij AG, Dutta R, Satija NK. Acclimatization to oxidative stress at high altitude. High Alt Med Biol. 2005;6(4):301–10. 10.1089/ham.2005.6.301 16351564

[34] Peden DB, Hohman R, Brown ME, Mason RT, Berkebile C, Fales HM, et al. Uric acid is a major antioxidant in human nasal airway secretions. Proc Natl Acad Sci. 1990;87(19):7638–42. 10.1073/pnas.87.19.7638 PMC54803 2217195

[35] Van Der Vliet A, O’Neill CA, Cross CE, Koostra JM, Volz WG, Halliwell B, et al. Determination of low-molecular-mass antioxidant concentrations in human respiratory tract lining fluids. Am J Physiol Lung Cell Mol Physiol. 1999;276(2)L289–96. 10.1152/ajplung.1999.276.2.L289 9950891

[36] Woolcott OO, Ader M, Bergman RN. Glucose homeostasis during short-term and prolonged exposure to high altitudes. Endocr Rev. 2015;36(2):149–73. 10.1210/er.2014-1063 PMC4399271 25675133

[37] Becher T, Palanisamy S, Kramer DJ, Eljalby M, Marx SJ, Wibmer AG, et al. Brown adipose tissue is associated with cardiometabolic health. Nat Med. 2021;27(1):58–65. 10.1038/s41591-020-1126-7 PMC8461455 33398160

[38] Cao Q, Jing J, Cui X, Shi H, Xue B. Sympathetic nerve innervation is required for beigeing in white fat. Physiol Rep. 2019;7(6):e14031. 10.14814/phy2.14031 PMC6418318 30873754

[39] Kazak L, Chouchani ET, Jedrychowski MP, Erickson BK, Shinoda K, Cohen P, et al. A creatine-driven substrate cycle enhances energy expenditure and thermogenesis in beige fat. Cell. 2015;163(3):643–55. 10.1016/j.cell.2015.09.035 PMC4656041 26496606

[40] Ikeda K, Kang Q, Yoneshiro T, Camporez JP, Maki H, Homma M, et al. UCP1-independent signaling involving SERCA2b-mediated calcium cycling regulates beige fat thermogenesis and systemic glucose homeostasis. Nat Med. 2017;23(12):1454–65. 29131158 10.1038/nm.4429 PMC5727902

[41] van Beek S, Hashim D, Bengtsson T, Hoeks J. Physiological and molecular mechanisms of cold-induced improvements in glucose homeostasis in humans beyond brown adipose tissue. Int J Obes. 2023;47(5):338–47. 10.1038/s41366-023-01270-z 36774412

[42] Rowland LA, Bal NC, Periasamy M. The role of skeletal-muscle-based thermogenic mechanisms in vertebrate endothermy. Biol Rev. 2015;90(4):1279–97. 10.1111/brv.12157 PMC4854186 25424279

[43] Scheele C, Wolfrum C. Brown adipose crosstalk in tissue plasticity and human metabolism. Endocr Rev. 2020;41(1):53–65. 10.1210/endrev/bnz007 PMC7006230 31638161

[44] Julian CG, Moore LG. Human genetic adaptation to high altitude: evidence from the Andes. Genes. 2019;10(2):150. 10.3390/genes10020150 PMC6410003 30781443

[45] Dong M, An K, Mao L. High levels of uric acid inhibit BAT thermogenic capacity through regulation of AMPK. Am J Physiol Endocrinol Metab. 2023;325(4):E376–89. 10.1152/ajpendo.00092.2023 PMC10642990 37732807

[46] Kotzbeck P, Giordano A, Mondini E, Murano I, Severi I, Venema W, et al. Brown adipose tissue whitening leads to brown adipocyte death and adipose tissue inflammation. J Lipid Res. 2018;59(5):784–94. 10.1194/jlr.M079665 PMC5928436 29599420

[47] Qiu J, Moore JH, Darabos C. Studying the genetics of complex disease with ancestry-specific human phenotype networks: the case of type 2 diabetes in East Asian populations. Genet Epidemiol. 2016;40(4):293–303. 10.1002/gepi.21964 PMC5071667 27061195

[48] Sniderman AD, Bhopal R, Prabhakaran D, Sarrafzadegan N, Tchernof A. Why might South Asians be so susceptible to central obesity and its atherogenic consequences? The adipose tissue overflow hypothesis. Int J Epidemio. 2007;36(1):220–5. 10.1093/ije/dyl245 17510078

[49] Tolla M. Human teeth characteristics and its development during Holocene time until current era in the lowland part of Papua Indonesia (Doctoral dissertation). [Berlin]: Freie Universität Berlin; 2024. Available from: https://refubium.fuberlin.de/bitstream/handle/fub188/45096/Dissertation_Marlin_Tolla_2024.pdf?sequence=3&isAllowed=y . Cited 2025 Apr 4.

[50] Kayser M, Lao O, Saar K, Brauer S, Wang X, Nürnberg P, et al. Genome-wide analysis indicates more Asian than Melanesian ancestry of Polynesians. Am J Hum Genet. 2008;82(1):194–8. 10.1016/j.ajhg.2007.09.010 PMC2253960 18179899

[51] Gaffney D, Summerhayes GR, Ford A, Scott JM, Denham T, Field J, et al. Earliest pottery on New Guinea mainland reveals Austronesian influences in highland environments 3000 years ago. PLoS One. 2015;10(9):e0134497. 10.1371/journal.pone.0134497 PMC4557931 26331310

[52] Yang HC, Chen CW, Lin YT, Chu SK. Genetic ancestry plays a central role in population pharmacogenomics. Commun Biol. 2021;4(1):171. 10.1038/s42003-021-01681-6 PMC7864978 33547344

[53] Roman Y, Tiirikainen M, Prom-Wormley E. The prevalence of the gout-associated polymorphism rs2231142 G>T in ABCG2 in a pregnant female Filipino cohort. Clinical rheumatology. 2020;39(8):2387–92. Available from: 10.1007/s10067-020-04994-9. 10.1007/s10067-020-04994-9 32107664

[54] Alghubayshi A, Edelman A, Alrajeh K, Roman Y. Genetic assessment of hyperuricemia and gout in Asian, Native Hawaiian, and Pacific Islander subgroups of pregnant women: biospecimens repository cross-sectional study. BMC Rheumatol. 2022;6:1–3. 10.1186/s41927-021-00239-7 PMC8734301 34986901

