# Supplementary material for: Anthropometric and metabolic differences and distribution of ABCG2 rs2231142 variant between lowland and highland Papuans in West Papua, Indonesia
Source: J Physiol Anthropol. 2025 May 20;44:14. doi: 10.1186/s40101-025-00394-7 (PMC12090604; doi:10.1186/s40101-025-00394-7)
Supplement: Supplementary file 1 — Additional file 1. Tests of Normality Shapiro–Wilk Before and After Data Transform & Mean Differences Tests: Highland and Lowland/Coast Men. [file 40101_2025_394_MOESM1_ESM.docx]

**Additional file 1**

**Tests of Normality Shapiro-Wilk Before and After Data Transform & Mean Differences Tests: Highland and Lowland/Coastal Men**

| **Original Data** | | | | | **Original & Transform Data** | | | | |
| --- | --- | --- | --- | --- | --- | --- | --- | --- | --- |
| **Variables** | **Category** | **Statistic** | **df** | **Sig.** | **Variables** | **Category** | **Statistic** | **df** | **Sig.** |
| Age | Highland | .855 | 40 | .000 | T_Age | Highland | .918 | 40 | .007 |
|  | Lowland | .968 | 45 | .238 |  | Lowland | .949 | 45 | .049 |
| BW | Highland | .953 | 40 | .094 | T_BW | Highland | .965 | 40 | .250 |
|  | Lowland | .885 | 45 | .000 |  | Lowland | .970 | 45 | .286 |
| BH | Highland | .959 | 40 | .153 | BH | Highland | .959 | 40 | .153 |
|  | Lowland | .955 | 45 | .076 |  | Lowland | .955 | 45 | .076 |
| BMI | Highland | .934 | 40 | .022 | T_BMI | Highland | .966 | 40 | .258 |
|  | Lowland | .846 | 45 | .000 |  | Lowland | .970 | 45 | .292 |
| WC | Highland | .922 | 40 | .009 | T_WC | Highland | .958 | 40 | .147 |
|  | Lowland | .891 | 45 | .001 |  | Lowland | .960 | 45 | .122 |
| HC | Highland | .976 | 40 | .552 | T_HC | Highland | .961 | 40 | .177 |
|  | Lowland | .911 | 45 | .002 |  | Lowland | .963 | 45 | .162 |
| WHR | Highland | .974 | 40 | .493 | T_WHR | Highland | .965 | 40 | .249 |
|  | Lowland | .947 | 45 | .038 |  | Lowland | .970 | 45 | .292 |
| WHtR | Highland | .959 | 40 | .152 | T_WHtR | Highland | .965 | 40 | .256 |
|  | Lowland | .924 | 45 | .006 |  | Lowland | .970 | 45 | .293 |
| Biceps | Highland | .732 | 40 | .000 | T_Biceps | Highland | .743 | 40 | .000 |
|  | Lowland | .845 | 45 | .000 |  | Lowland | .908 | 45 | .002 |
| Triceps | Highland | .896 | 40 | .001 | T_Triceps | Highland | .913 | 40 | .005 |
|  | Lowland | .864 | 45 | .000 |  | Lowland | .942 | 45 | .026 |
| Subscapular | Highland | .778 | 40 | .000 | T_Subscapular | Highland | .906 | 40 | .003 |
|  | Lowland | .928 | 45 | .008 |  | Lowland | .919 | 45 | .004 |
| Suprailiac | Highland | .894 | 40 | .001 | T_Suprailiac | Highland | .932 | 40 | .019 |
|  | Lowland | .946 | 45 | .036 |  | Lowland | .930 | 45 | .009 |
| BF(%) | Highland | .979 | 40 | .648 | BF(%) | Highland | .979 | 40 | .648 |
|  | Lowland | .966 | 45 | .210 |  | Lowland | .966 | 45 | .210 |
| TF | Highland | .960 | 40 | .171 | TF | Highland | .960 | 40 | .171 |
|  | Lowland | .961 | 45 | .134 |  | Lowland | .961 | 45 | .134 |
| UA | Highland | .965 | 40 | .243 | T_UA | Highland | .963 | 40 | .204 |
|  | Lowland | .886 | 45 | .000 |  | Lowland | .965 | 45 | .187 |
| TC | Highland | .945 | 40 | .052 | T_TC | Highland | .963 | 40 | .214 |
|  | Lowland | .883 | 45 | .000 |  | Lowland | .952 | 45 | .061 |
| FBG | Highland | .960 | 40 | .165 | T_FBG | Highland | .961 | 40 | .187 |
|  | Lowland | .712 | 45 | .000 |  | Lowland | .949 | 45 | .049 |
| RBG | Highland | .961 | 40 | .186 | T_RBG | Highland | .962 | 40 | .202 |
|  | Lowland | .925 | 45 | .007 |  | Lowland | .967 | 45 | .230 |
| SBP | Highland | .964 | 40 | .231 | SBP | Highland | .964 | 40 | .231 |
|  | Lowland | .969 | 45 | .265 |  | Lowland | .969 | 45 | .265 |
| DBP | Highland | .979 | 40 | .670 | DBP | Highland | .979 | 40 | .670 |
|  | Lowland | .970 | 45 | .282 |  | Lowland | .970 | 45 | .282 |
