# Supplementary material for: Anthropometric and metabolic differences and distribution of ABCG2 rs2231142 variant between lowland and highland Papuans in West Papua, Indonesia
Source: J Physiol Anthropol. 2025 May 20;44:14. doi: 10.1186/s40101-025-00394-7 (PMC12090604; doi:10.1186/s40101-025-00394-7)
Supplement: Supplementary file 2 — Additional file 2. Tests of Normality Shapiro–Wilk Before and After Data Transform tests & Mean Differences Tests: Highland and Lowland/Coast Women. [file 40101_2025_394_MOESM2_ESM.docx]

**Additional file 2**

**Tests of Normality Shapiro-Wilk Before and After Data Transform tests & Mean Differences Tests: Highland and Lowland/Coastal Women**

| **Original Data** | | | | | **Original & Transform Data** | | | | |
| --- | --- | --- | --- | --- | --- | --- | --- | --- | --- |
| **Variables** | **Category** | **Statistic** | **df** | **Sig.** | **Variables** | **Category** | **Statistic** | **df** | **Sig.** |
| Age | Highland | .869 | 25 | .004 | T_Age | Highland | .933 | 25 | .100 |
|  | Lowland | .854 | 33 | .000 |  | Lowland | .921 | 33 | .020 |
| BW | Highland | .940 | 25 | .148 | T_BW | Highland | .942 | 25 | .166 |
|  | Lowland | .866 | 33 | .001 |  | Lowland | .956 | 33 | .202 |
| BH | Highland | .926 | 25 | .072 | BH | Highland | .926 | 25 | .072 |
|  | Lowland | .960 | 33 | .260 |  | Lowland | .960 | 33 | .260 |
| BMI | Highland | .941 | 25 | .160 | T_BMI | Highland | .942 | 25 | .166 |
|  | Lowland | .858 | 33 | .001 |  | Lowland | .957 | 33 | .212 |
| WC | Highland | .977 | 25 | .811 | T_WC | Highland | .939 | 25 | .144 |
|  | Lowland | .927 | 33 | .029 |  | Lowland | .948 | 33 | .119 |
| HC | Highland | .975 | 25 | .764 | T_HC | Highland | .937 | 25 | .128 |
|  | Lowland | .920 | 33 | .019 |  | Lowland | .952 | 33 | .148 |
| WHR | Highland | .960 | 25 | .422 | T_WHR | Highland | .929 | 25 | .083 |
|  | Lowland | .929 | 33 | .032 |  | Lowland | .946 | 33 | .104 |
| WHtR | Highland | .974 | 25 | .746 | T_WHtR | Highland | .935 | 25 | .116 |
|  | Lowland | .917 | 33 | .015 |  | Lowland | .946 | 33 | .104 |
| Biceps | Highland | .908 | 25 | .028 | T_Biceps | Highland | .872 | 25 | .005 |
|  | Lowland | .920 | 33 | .019 |  | Lowland | .928 | 33 | .030 |
| Triceps | Highland | .877 | 25 | .006 | T_Triceps | Highland | .923 | 25 | .059 |
|  | Lowland | .940 | 33 | .069 |  | Lowland | .944 | 33 | .088 |
| Subscapular | Highland | .904 | 25 | .022 | T_ Subscapular | Highland | .922 | 25 | .058 |
|  | Lowland | .911 | 33 | .010 |  | Lowland | .886 | 33 | .002 |
| Suprailiac | Highland | .891 | 25 | .011 | T_ Suprailiac | Highland | .932 | 25 | .099 |
|  | Lowland | .778 | 33 | .000 |  | Lowland | .914 | 33 | .013 |
| BF(%) | Highland | .978 | 25 | .840 | T_BF | Highland | .941 | 25 | .160 |
|  | Lowland | .873 | 33 | .001 |  | Lowland | .952 | 33 | .156 |
| TF | Highland | .961 | 25 | .445 | T_TF | Highland | .942 | 25 | .166 |
|  | Lowland | .931 | 33 | .037 |  | Lowland | .957 | 33 | .212 |
| UA | Highland | .802 | 25 | .000 | T_UA | Highland | .941 | 25 | .158 |
|  | Lowland | .940 | 33 | .066 |  | Lowland | .954 | 33 | .172 |
| TC | Highland | .757 | 25 | .000 | T_TC | Highland | .941 | 25 | .153 |
|  | Lowland | .840 | 33 | .000 |  | Lowland | .956 | 33 | .202 |
| FBG | Highland | .948 | 25 | .229 | FBG | Highland | .948 | 25 | .229 |
|  | Lowland | .953 | 33 | .159 |  | Lowland | .953 | 33 | .159 |
| RBG | Highland | .972 | 25 | .683 | RBG | Highland | .972 | 25 | .683 |
|  | Lowland | .972 | 33 | .528 |  | Lowland | .972 | 33 | .528 |
| SBP | Highland | .954 | 25 | .305 | SBP | Highland | .954 | 25 | .305 |
|  | Lowland | .957 | 33 | .216 |  | Lowland | .957 | 33 | .216 |
| DBP | Highland | .977 | 25 | .811 | T_DBP | Highland | .939 | 25 | .137 |
|  | Lowland | .931 | 33 | .038 |  | Lowland | .930 | 33 | .035 |
