# Supplementary material for: Anthropometric and metabolic differences and distribution of ABCG2 rs2231142 variant between lowland and highland Papuans in West Papua, Indonesia
Source: J Physiol Anthropol. 2025 May 20;44:14. doi: 10.1186/s40101-025-00394-7 (PMC12090604; doi:10.1186/s40101-025-00394-7)
Supplement: Supplementary file 3 — Additional file 3. Summary Tables of Normality and Linearity Tests for Correlation Analysis Between Clinical and Anthropometric Data of Lowland Highland Men. [file 40101_2025_394_MOESM3_ESM.docx]

**Additional file 3**

**Summary Tables of Normality and Linearity Tests for Correlation Analysis Between Clinical and Anthropometric Data of Lowland/Coastal Highland Men**

| **Variable 1** | **Variable 2** | **Fulfillment of Statistical Test Requirements** | | **Correlation Analysis** |
| --- | --- | --- | --- | --- |
|  |  | **Normality Test** | **Linearity**  **Test** |  |
| Uric Acid | Age | Yes | Yes | Pearson |
|  | Body Weight | Yes | Yes | Pearson |
|  | Body Height | Yes | Yes | Pearson |
|  | Body Mass Index | Yes | No | Spearman |
|  | Waist Circumference | Yes | Yes | Pearson |
|  | Hip Circumference | Yes | Yes | Pearson |
|  | Waist to Hip Ratio | Yes | Yes | Pearson |
|  | Waist-to-Height Ratio | Yes | Yes | Pearson |
|  | Triceps | Yes | Yes | Pearson |
|  | Body Fat (%) | Yes | Yes | Pearson |
|  | Total Fat | Yes | Yes | Pearson |
| Total Cholesterol | Age | Yes | Yes | Pearson |
|  | Body Weight | Yes | Yes | Pearson |
|  | Body Height | Yes | Yes | Pearson |
|  | Body Mass Index | Yes | No | Spearman |
|  | Waist Circumference | Yes | Yes | Pearson |
|  | Hip Circumference | Yes | Ye | Pearson |
|  | Waist to Hip Ratio | Yes | Yes | Pearson |
|  | Waist-to-Height Ratio | Yes | Yes | Pearson |
|  | Triceps | Yes | Ye | Pearson |
|  | Body Fat (%) | Yes | Yes | Pearson |
|  | Total Fat | Yes | Yes | Pearson |
| Fasting Blood Glucose | Age | Yes | Yes | Pearson |
|  | Body Weight | Yes | Yes | Pearson |
|  | Body Height | Yes | Yes | Pearson |
|  | Body Mass Index | Yes | No | Spearman |
|  | Waist Circumference | Yes | Yes | Pearson |
|  | Hip Circumference | Yes | Yes | Pearson |
|  | Waist to Hip Ratio | Yes | Yes | Pearson |
|  | Waist-to-Height Ratio | Yes | Yes | Pearson |
|  | Triceps | Yes | Yes | Pearson |
|  | Body Fat (%) | Yes | Yes | Pearson |
|  | Total Fat | Yes | Yes | Pearson |
| Random Blood Glucose | Age | Yes | Yes | Pearson |
|  | Body Weight | Yes | Yes | Pearson |
|  | Body Height | Yes | Yes | Pearson |
|  | Body Mass Index | Yes | No | Spearman |
|  | Waist Circumference | Yes | Yes | Pearson |
|  | Hip Circumference | Yes | Yes | Pearson |
|  | Waist to Hip Ratio | Yes | Yes | Pearson |
|  | Waist-to-Height Ratio | Yes | Yes | Pearson |
|  | Triceps | Yes | Yes | Pearson |
|  | Body Fat (%) | Yes | Yes | Pearson |
|  | Total Fat | Yes | Yes | Pearson |
| Systolic Blood Pressure | Age | Yes | Yes | Pearson |
|  | Body Weight | Yes | Yes | Pearson |
|  | Body Height | Yes | Yes | Pearson |
|  | Body Mass Index | Yes | No | Spearman |
|  | Waist Circumference | Yes | Yes | Pearson |
|  | Hip Circumference | Yes | Yes | Pearson |
|  | Waist to Hip Ratio | Yes | Yes | Pearson |
|  | Waist-to-Height Ratio | Yes | Yes | Pearson |
|  | Triceps | Yes | Yes | Pearson |
|  | Body Fat (%) | Yes | Yes | Pearson |
|  | Total Fat | Yes | Yes | Pearson |
| Diastolic Blood Pressure | Age | Yes | Yes | Pearson |
|  | Body Weight | Yes | Yes | Pearson |
|  | Body Height | Yes | Yes | Pearson |
|  | Body Mass Index | Yes | No | Spearman |
|  | Waist Circumference | Yes | Yes | Pearson |
|  | Hip Circumference | Yes | Yes | Pearson |
|  | Waist to Hip Ratio | Yes | Yes | Pearson |
|  | Waist-to-Height Ratio | Yes | Yes | Pearson |
|  | Triceps | Yes | Yes | Pearson |
|  | Body Fat (%) | Yes | Yes | Pearson |
|  | Total Fat | Yes | Yes | Pearson |

**Summary Table of Normality and Linearity Tests for Correlation Analysis Between Clinical and Anthropometric Data of Lowland Men**

| **Variable 1** | **Variable 2** | **Fulfillment of Statistical Test Requirements*** | | **Correlation Analysis** |
| --- | --- | --- | --- | --- |
|  |  | **Normality Test** | **Linearity Test** |  |
| Uric Acid | Age | Yes | Yes | Pearson |
|  | Body Weight | Yes | Yes | Pearson |
|  | Body Height | Yes | Yes | Pearson |
|  | Body Mass Index | No | No | Spearman |
|  | Waist Circumference | No | No | Spearman |
|  | Hip Circumference | No | No | Spearman |
|  | Waist to Hip Ratio | No | No | Spearman |
|  | Waist-to-Height Ratio | No | No | Spearman |
|  | Triceps | No | No | Spearman |
|  | Body Fat (%) | Yes | Yes | Pearson |
|  | Total Fat | Yes | No | Spearman |
| Total Cholesterol | Age | Yes | Yes | Pearson |
|  | Body Weight | Yes | No | Spearman |
|  | Body Height | Yes | Yes | Pearson |
|  | Body Mass Index | No | No | Spearman |
|  | Waist Circumference | No | No | Spearman |
|  | Hip Circumference | No | No | Spearman |
|  | Waist to Hip Ratio | No | No | Spearman |
|  | Waist-to-Height Ratio | No | No | Spearman |
|  | Triceps | No | No | Spearman |
|  | Body Fat (%) | Yes | Yes | Pearson |
|  | Total Fat | Yes | Yes | Pearson |
| Fasting Blood Glucose | Age | Yes | Yes | Pearson |
|  | Body Weight | Yes | Yes | Pearson |
|  | Body Height | Yes | Yes | Pearson |
|  | Body Mass Index | No | No | Spearman |
|  | Waist Circumference | No | No | Spearman |
|  | Hip Circumference | No | No | Spearman |
|  | Waist to Hip Ratio | No | No | Spearman |
|  | Waist-to-Height Ratio | No | No | Spearman |
|  | Triceps | No | No | Spearman |
|  | Body Fat (%) | Yes | Yes | Pearson |
|  | Total Fat | Yes | Yes | Pearson |
| Random Blood Glucose | Age | Yes | Yes | Pearson |
|  | Body Weight | Yes | Yes | Pearson |
|  | Body Height | Yes | Yes | Pearson |
|  | Body Mass Index | No | No | Spearman |
|  | Waist Circumference | No | No | Spearman |
|  | Hip Circumference | No | No | Spearman |
|  | Waist to Hip Ratio | No | No | Spearman |
|  | Waist-to-Height Ratio | No | No | Spearman |
|  | Triceps | No | No | Spearman |
|  | Body Fat (%) | Yes | Yes | Pearson |
|  | Total Fat | Yes | Yes | Pearson |
| Systolic Blood Pressure | Age | Yes | Yes | Pearson |
|  | Body Weight | Yes | Yes | Pearson |
|  | Body Height | Yes | Yes | Pearson |
|  | Body Mass Index | Yes | No | Spearman |
|  | Waist Circumference | Yes | Yes | Pearson |
|  | Hip Circumference | Yes | Yes | Pearson |
|  | Waist to Hip Ratio | Yes | Yes | Pearson |
|  | Waist-to-Height Ratio | Yes | No | Spearman |
|  | Triceps | Yes | Yes | Pearson |
|  | Body Fat (%) | Yes | Yes | Pearson |
|  | Total Fat | Yes | Yes | Pearson |
| Diastolic Blood Pressure | Age | Yes | Yes | Pearson |
|  | Body Weight | Yes | Yes | Pearson |
|  | Body Height | Yes | Yes | Pearson |
|  | Body Mass Index | Yes | No | Spearman |
|  | Waist Circumference | Yes | Yes | Pearson |
|  | Hip Circumference | Yes | Yes | Pearson |
|  | Waist to Hip Ratio | Yes | Yes | Pearson |
|  | Waist-to-Height Ratio | Yes | No | Spearman |
|  | Triceps | Yes | Yes | Pearson |
|  | Body Fat (%) | Yes | Yes | Pearson |
|  | Total Fat | Yes | Yes | Pearson |

**Summary Tables of Normality and Linearity Tests for Correlation Analysis Between Clinical and Anthropometric Data of Highland Women**

| **Variable 1** | **Variable 2** | **Fulfillment of Statistical Test Requirements** | | **Correlation**  **Analysis** |
| --- | --- | --- | --- | --- |
|  |  | **Normality Test** | **Linearity**  **Test** |  |
| Uric Acid | Age | No | No | Spearman |
|  | Body Weight | Yes | Yes | Pearson |
|  | Body Height | Yes | Yes | Pearson |
|  | Body Mass Index | Yes | Yes | Pearson |
|  | Waist Circumference | Yes | Yes | Pearson |
|  | Hip Circumference | Yes | Yes | Pearson |
|  | Waist to Hip Ratio | Yes | Yes | Pearson |
|  | Waist-to-Height Ratio | Yes | Yes | Pearson |
|  | Triceps | No | No | Spearman |
|  | Body Fat (%) | Yes | Yes | Pearson |
|  | Total Fat | Yes | Yes | Pearson |
| Total Cholesterol | Age | No | No | Spearman |
|  | Body Weight | Yes | Yes | Pearson |
|  | Body Height | Yes | Yes | Pearson |
|  | Body Mass Index | Yes | Yes | Pearson |
|  | Waist Circumference | Yes | Yes | Pearson |
|  | Hip Circumference | Yes | Yes | Pearson |
|  | Waist to Hip Ratio | Yes | No | Spearman |
|  | Waist-to-Height Ratio | Yes | Yes | Pearson |
|  | Triceps | No | No | Spearman |
|  | Body Fat (%) | Yes | Yes | Pearson |
|  | Total Fat | Yes | Yes | Pearson |
| Fasting Blood Glucose | Age | Yes | Yes | Pearson |
|  | Body Weight | Yes | Yes | Pearson |
|  | Body Height | Yes | No | Spearman |
|  | Body Mass Index | Yes | Yes | Pearson |
|  | Waist Circumference | Yes | Yes | Pearson |
|  | Hip Circumference | Yes | Yes | Pearson |
|  | Waist to Hip Ratio | Yes | Yes | Pearson |
|  | Waist-to-Height Ratio | Yes | Yes | Pearson |
|  | Triceps | Yes | Yes | Pearson |
|  | Body Fat (%) | Yes | Yes | Pearson |
|  | Total Fat | Yes | Yes | Pearson |
| Random Blood Glucose | Age | Yes | Yes | Pearson |
|  | Body Weight | Yes | Yes | Pearson |
|  | Body Height | Yes | Yes | Pearson |
|  | Body Mass Index | Yes | Yes | Pearson |
|  | Waist Circumference | Yes | Yes | Pearson |
|  | Hip Circumference | Yes | Yes | Pearson |
|  | Waist to Hip Ratio | Yes | Yes | Pearson |
|  | Waist-to-Height Ratio | Yes | Yes | Pearson |
|  | Triceps | Yes | Yes | Pearson |
|  | Body Fat (%) | Yes | Yes | Pearson |
|  | Total Fat | Yes | Yes | Pearson |
| Systolic Blood Pressure | Age | Yes | Yes | Pearson |
|  | Body Weight | Yes | No | Spearman |
|  | Body Height | Yes | Yes | Pearson |
|  | Body Mass Index | Yes | Yes | Pearson |
|  | Waist Circumference | Yes | Yes | Pearson |
|  | Hip Circumference | Yes | Yes | Pearson |
|  | Waist to Hip Ratio | Yes | Yes | Pearson |
|  | Waist-to-Height Ratio | Yes | Yes | Pearson |
|  | Triceps | Yes | Yes | Pearson |
|  | Body Fat (%) | Yes | Yes | Pearson |
|  | Total Fat | Yes | Yes | Pearson |
| Diastolic Blood Pressure | Age | Yes | Yes | Pearson |
|  | Body Weight | Yes | Yes | Pearson |
|  | Body Height | Yes | No | Spearman |
|  | Body Mass Index | Yes | Yes | Pearson |
|  | Waist Circumference | Yes | Yes | Pearson |
|  | Hip Circumference | Yes | Yes | Pearson |
|  | Waist to Hip Ratio | Yes | Yes | Pearson |
|  | Waist-to-Height Ratio | Yes | Yes | Pearson |
|  | Triceps | Yes | Yes | Pearson |
|  | Body Fat (%) | Yes | Yes | Pearson |
|  | Total Fat | Yes | Yes | Pearson |

**Summary Tables of Normality and Linearity Tests for Correlation Analysis Between Clinical and Anthropometric Data of Lowland Women**

| **Variable 1** | **Variable 2** | **Fulfillment of Statistical Test Requirements** | | **Correlation Analysis** |
| --- | --- | --- | --- | --- |
|  |  | **Normality Test** | **Linearity**  **Test** |  |
| Uric Acid | Age | Yes | Yes | Pearson |
|  | Body Weight | Yes | Yes | Pearson |
|  | Body Height | Yes | Yes | Pearson |
|  | Body Mass Index | Yes | No | Spearman |
|  | Waist Circumference | Yes | Yes | Pearson |
|  | Hip Circumference | Yes | Yes | Pearson |
|  | Waist to Hip Ratio | Yes | Yes | Pearson |
|  | Waist-to-Height Ratio | Yes | Yes | Pearson |
|  | Triceps | Yes | Yes | Pearson |
|  | Body Fat (%) | Yes | Yes | Pearson |
|  | Total Fat | Yes | No | Spearman |
| Total Cholesterol | Age | No | No | Spearman |
|  | Body Weight | No | No | Spearman |
|  | Body Height | Yes | Yes | Pearson |
|  | Body Mass Index | No | No | Spearman |
|  | Waist Circumference | No | No | Spearman |
|  | Hip Circumference | No | No | Spearman |
|  | Waist to Hip Ratio | No | No | Spearman |
|  | Waist-to-Height Ratio | No | No | Spearman |
|  | Triceps | Yes | Yes | Pearson |
|  | Body Fat (%) | No | No | Spearman |
|  | Total Fat | No | No | Spearman |
| Fasting Blood Glucose | Age | Yes | No | Spearman |
|  | Body Weight | Yes | Yes | Pearson |
|  | Body Height | Yes | Yes | Pearson |
|  | Body Mass Index | Yes | No | Spearman |
|  | Waist Circumference | Yes | Yes | Pearson |
|  | Hip Circumference | Yes | Yes | Pearson |
|  | Waist to Hip Ratio | Yes | Yes | Pearson |
|  | Waist-to-Height Ratio | Yes | Yes | Pearson |
|  | Triceps | Yes | Yes | Pearson |
|  | Body Fat (%) | Yes | Yes | Pearson |
|  | Total Fat | Yes | No | Spearman |
| Random Blood Glucose | Age | Yes | Yes | Pearson |
|  | Body Weight | Yes | Yes | Pearson |
|  | Body Height | Yes | Yes | Pearson |
|  | Body Mass Index | Yes | No | Spearman |
|  | Waist Circumference | Yes | Yes | Pearson |
|  | Hip Circumference | Yes | Yes | Pearson |
|  | Waist to Hip Ratio | Yes | Yes | Pearson |
|  | Waist-to-Height Ratio | Yes | Yes | Pearson |
|  | Triceps | Yes | Yes | Pearson |
|  | Body Fat (%) | Yes | Yes | Pearson |
|  | Total Fat | Yes | No | Spearman |
| Systolic Blood Pressure | Age | Yes | Yes | Pearson |
|  | Body Weight | Yes | Yes | Pearson |
|  | Body Height | Yes | Yes | Pearson |
|  | Body Mass Index | Yes | No | Spearman |
|  | Waist Circumference | Yes | Yes | Pearson |
|  | Hip Circumference | Yes | Yes | Pearson |
|  | Waist to Hip Ratio | Yes | Yes | Pearson |
|  | Waist-to-Height Ratio | Yes | No | Spearman |
|  | Triceps | Yes | Yes | Pearson |
|  | Body Fat (%) | Yes | No | Spearman |
|  | Total Fat | Yes | No | Spearman |
| Diastolic Blood Pressure | Age | No | No | Spearman |
|  | Body Weight | No | No | Spearman |
|  | Body Height | Yes | Yes | Pearson |
|  | Body Mass Index | No | No | Spearman |
|  | Waist Circumference | No | No | Spearman |
|  | Hip Circumference | No | No | Spearman |
|  | Waist to Hip Ratio | No | No | Spearman |
|  | Waist-to-Height Ratio | No | No | Spearman |
|  | Triceps | Yes | Yes | Pearson |
|  | Body Fat (%) | No | No | Spearman |
|  | Total Fat | NO | No | Spearman |
