# Supplementary material for: Anthropometric and metabolic differences and distribution of ABCG2 rs2231142 variant between lowland and highland Papuans in West Papua, Indonesia
Source: J Physiol Anthropol. 2025 May 20;44:14. doi: 10.1186/s40101-025-00394-7 (PMC12090604; doi:10.1186/s40101-025-00394-7)
Supplement: Supplementary file 4 — Additional file 4. Comparison of ABCG2 rs22231142 Variant Against Anthropometry of Papuan Participants in Salatiga. [file 40101_2025_394_MOESM4_ESM.docx]

**Additional file 4**

| Comparison of *ABCG2 rs22231142* Variant Against Anthropometry of Papuan Participants in Salatiga | | | | | | | | | | |
| --- | --- | --- | --- | --- | --- | --- | --- | --- | --- | --- |
| **Sex** | **Phenotype** | **Genotype** | **Lowland*** | | | ***p-value*** | **Highland**** | | | ***p-value*** |
|  |  |  | **Mean (± SD)** | | |  | **Mean (± SD)** | | |  |
| Men | BMI | GT | 22.99 | ± | 3.77 | 0.701 | 25.46 | ± | 2.43 | 0.752 |
|  |  | GG | 23.55 | ± | 4.87 |  | 25.88 | ± | 3.33 |  |
| Women | BMI | GT | 24.57 | ± | 5.45 | 0.951 | 24.60 | ± | 2.01 | 0.497 |
|  |  | GG | 24.45 | ± | 6.03 |  | 26.67 | ± | 4.13 |  |
| Men | WHR | GT | 0.86 | ± | 0.06 | 0.795 | 0.86 | ± | 0.03 | 0.067 |
|  |  | GG | 0.86 | ± | 0.04 |  | 0.88 | ± | 0.04 |  |
| WHR | WHR | GT | 0.82 | ± | 0.05 | 0.173 | 0.84 | ± | 0.06 | 0.450 |
|  |  | GG | 0.85 | ± | 0.07 |  | 0.86 | ± | 0.05 |  |
| Men | BF(%) | GT | 6.85 | ± | 3.81 | 0.702 | 6.37 | ± | 2.41 | 0.524 |
|  |  | GG | 6.38 | ± | 3.83 |  | 7.26 | ± | 3.46 |  |
| Women | BF(%) | GT | 9.29 | ± | 2.72 | 0.329 | 16.05 | ± | 3.46 | 0.556 |
|  |  | GG | 7.84 | ± | 4.97 |  | 18.29 | ± | 5.14 |  |
| * : Lowland Participants 76 (Men GG=30, GT =15 ; Women GG =16, GT=15) | | | | | | | | | | |
| ** : Highland Participants 64 (Men GG=33, GT =7; Women GG =22, GT=22) | | | | | | | | | | |
