# Supplementary material for: Anthropometric and metabolic differences and distribution of ABCG2 rs2231142 variant between lowland and highland Papuans in West Papua, Indonesia
Source: J Physiol Anthropol. 2025 May 20;44:14. doi: 10.1186/s40101-025-00394-7 (PMC12090604; doi:10.1186/s40101-025-00394-7)
Supplement: Supplementary file 5 — Additional file 5. Comparison of ABCG2 rs22231142 Variant Against Blood Chemistry and Clinical Data of Papuan Participants in Salatiga. [file 40101_2025_394_MOESM5_ESM.docx]

**Additional file 5**

| Comparison of *ABCG2* rs22231142 Variant Against Blood Chemistry and Clinical Data of Papuan Participants in Salatiga | | | | | | | | | | |
| --- | --- | --- | --- | --- | --- | --- | --- | --- | --- | --- |
| **Sex** | **Phenotype** | **Genotype** | **Lowland GG/GT** | | | | **Highland GG/GT** | | | |
|  |  |  | **Mean (± SD)** | | | ***p-value*** | **Mean (± SD)** | | | ***p-value*** |
| Men | UA (mg/dl) | GT | 5.7 | ± | 2.2 | 0.712 | 6.1 | ± | 1.2 | 0,414 |
|  |  | GG | 5.9 | ± | 1.6 |  | 6.6 | ± | 1.4 |  |
| Women | UA (mg/dl) | GT | 5.84 | ± | 1.50 | 0.732 | 7.9 | ± | 3.3 | 0.495 |
|  |  | GG | 6.03 | ± | 1.58 |  | 6.7 | ± | 2.2 |  |
| Men | TC (mg/dl) | GT | 160.1 | ± | 41.8 | 0.711 | 138.4 | ± | 35.2 | 0,112 |
|  |  | GG | 155.9 | ± | 32.7 |  | 164.2 | ± | 38.6 |  |
| Women | TC (mg/dl) | GT | 138.8 | ± | 37.9 | 0.313 | 146.0 | ± | 2.8 | 0.527 |
|  |  | GG | 154.1 | ± | 44.6 |  | 168.1 | ± | 47.6 |  |
| Men | FBG (mg/dl) | GT | 84.7 | ± | 14.6 | 0.475 | 83.29 | ± | 10.5 | 0.402 |
|  |  | GG | 87.4 | ± | 10.2 |  | 79.30 | ± | 11.4 |  |
| Women | FBG (mg/dl) | GT | 91.8 | ± | 9.2 | 0.040 | 71.0 | ± | 9.9 | 0.468 |
|  |  | GG | 84.7 | ± | 9.1 |  | 76.7 | ± | 10.5 |  |
| Men | RBG (mg/dl) | GT | 100.5 | ± | 10.5 | 0.299 | 93.7 | ± | 20.5 | 0.195 |
|  |  | GG | 107.3 | ± | 24.1 |  | 103.5 | ± | 17.4 |  |
| Women | RBG (mg/dl) | GT | 104.5 | ± | 20.6 | 0.443 | 91.0 | ± | 5.6 | 0.432 |
|  |  | GG | 99.2 | ± | 17.0 |  | 101.4 | ± | 18.0 |  |
| Men | SBP (mmHg) | GT | 123.4 | ± | 9.6 | 0.604 | 130.3 | ± | 15.6 | 0,036 |
|  |  | GG | 122.0 | ± | 7.5 |  | 120.8 | ± | 9.3 |  |
| Women | SBP (mmHg) | GT | 110.5 | ± | 10.4 | 0.099 | 100.0 | ± | 0.0 | 0.151 |
|  |  | GG | 116.4 | ± | 8.8 |  | 108.4 | ± | 7.8 |  |
| Men | DBP (mmHg) | GT | 81.4 | ± | 7.1 | 0.921 | 82.4 | ± | 5.4 | 0,420 |
|  |  | GG | 81.6 | ± | 7.5 |  | 79.8 | ± | 8.2 |  |
| Women | DBP (mmHg) | GT | 78.4 | ± | 7.4 | 0.377 | 66.5 | ± | 3.5 | 0.174 |
|  |  | GG | 80.9 | ± | 7.9 |  | 75.0 | ± | 8.4 |  |
| *p-value* significant<0.05  * Lowland Participants 76 (Men GG=30, GT =15 and Women GG =16, GT=15) | | | | | | | | | | |
| **Highland Participants 64 (Men GG=33, GT ; 7= and Women GG =22, GT=22) | | | | | | | | | | |
