# Supplementary material for: Anthropometric and metabolic differences and distribution of ABCG2 rs2231142 variant between lowland and highland Papuans in West Papua, Indonesia
Source: J Physiol Anthropol. 2025 May 20;44:14. doi: 10.1186/s40101-025-00394-7 (PMC12090604; doi:10.1186/s40101-025-00394-7)
Supplement: Supplementary file 6 — Additional file 6. Anthropometric and blood chemistry values of Papuan students from lowland and highland areas based on length of stay in Salatiga (average ± SD). [file 40101_2025_394_MOESM6_ESM.docx]

**Additional file 6**

Anthropometric and blood chemistry values ​​of Papuan students from lowland and highland areas based on length of stay in Salatiga (average±SD)

| **Lowland** | | | | | | | | |
| --- | --- | --- | --- | --- | --- | --- | --- | --- |
| **Sex** | **Local Exposure** | **BMI** | **WC‎/HC** | **BF (%)** | **UA** | **TC** | **FBG** | **RBG** |
| Men | <3 year (N= 15) | 21.15±1.7 | 0.85±0.04 | 10.5±3.21 | 4.8±0.8 | 152.6±36.6 | 85.3±8.3 | 98.9±16.8 |
|  | ≥3 year (N= 30) | 24.47±5.05 | 0.86±0.05 | 9.78±4.91 | 6.5±2 | 159.7±35.5 | 87.1±13.3 | 108.1±22 |
| Chi-Square Test | | 0.439 | 0.502 | 0.367 | 0.386 | 0.518 | 0.11 | 0.402 |
| Women | <3 year (N= 6) | 24.7±7.17 | 0.84±0.08 | 12.41±4.65 | 5.6±1.12 | 155.2±66.74 | 89.3±7.74 | 106.8±16.57 |
|  | ≥3 year (N= 27) | 24.17±5.31 | 0.83±0.06 | 14.17±3.57 | 5.9±1.6 | 143.5±33.81 | 88.5±10.3 | 100.3±18.66 |
| Chi-Square Test | | 0.37 | 0.091 | 0.298 | 0.632 | 0.448 | 0.516 | 0.621 |
| **Highland** | | | | | | | | |
| **Sex** | **Local Exposure** | **BMI** | **WC‎/HC** | **BF (%)** | **UA** | **TC** | **FBG** | **RBG** |
| Men | <3 year (N= 17) | 24.83±3.51 | 0.88±0.04 | 10.59±4.06 | 6.8±1.5 | 160.6±38.6 | 75.8±9.8 | 103.5±17 |
|  | ≥3 year (N= 23) | 27.73±3.64 | 0.87±0.04 | 25.78±8.47 | 6.7±2.2 | 160.2±50.4 | 77.7±10.6 | 99.9±19.8 |
| Chi-Square Test | | 0.52 | 0.796 | 0.129 | 0.537 | 0.585 | 0.716 | 0.426 |
| Women | <3 year (N= 16) | 26.28±2.36 | 0.87±0.04 | 10.36±3.42 | 6.4±1.49 | 168.9±40.15 | 84.6±12.26 | 103.6±20.32 |
|  | ≥3 year (N= 9) | 24.35±3.65 | 0.86±0.06 | 22.31±9.05 | 6.9±1.7 | 160.7±22.1 | 74±9.2 | 98.9±6.4 |
| Chi-Square Test | | 0.406 | 0.436 | 0.356 | 0.538 | 0.618 | 0.242 | 0.404 |
